# Supplementary figures and images for: The relationship between diet, plasma glucose, and cancer prevalence across vertebrates
Source: bioRxiv. 2023 Aug 2:2023.07.31.551378. Preprint. [Version 1] doi: 10.1101/2023.07.31.551378 (PMC10418110; doi:10.1101/2023.07.31.551378)

A

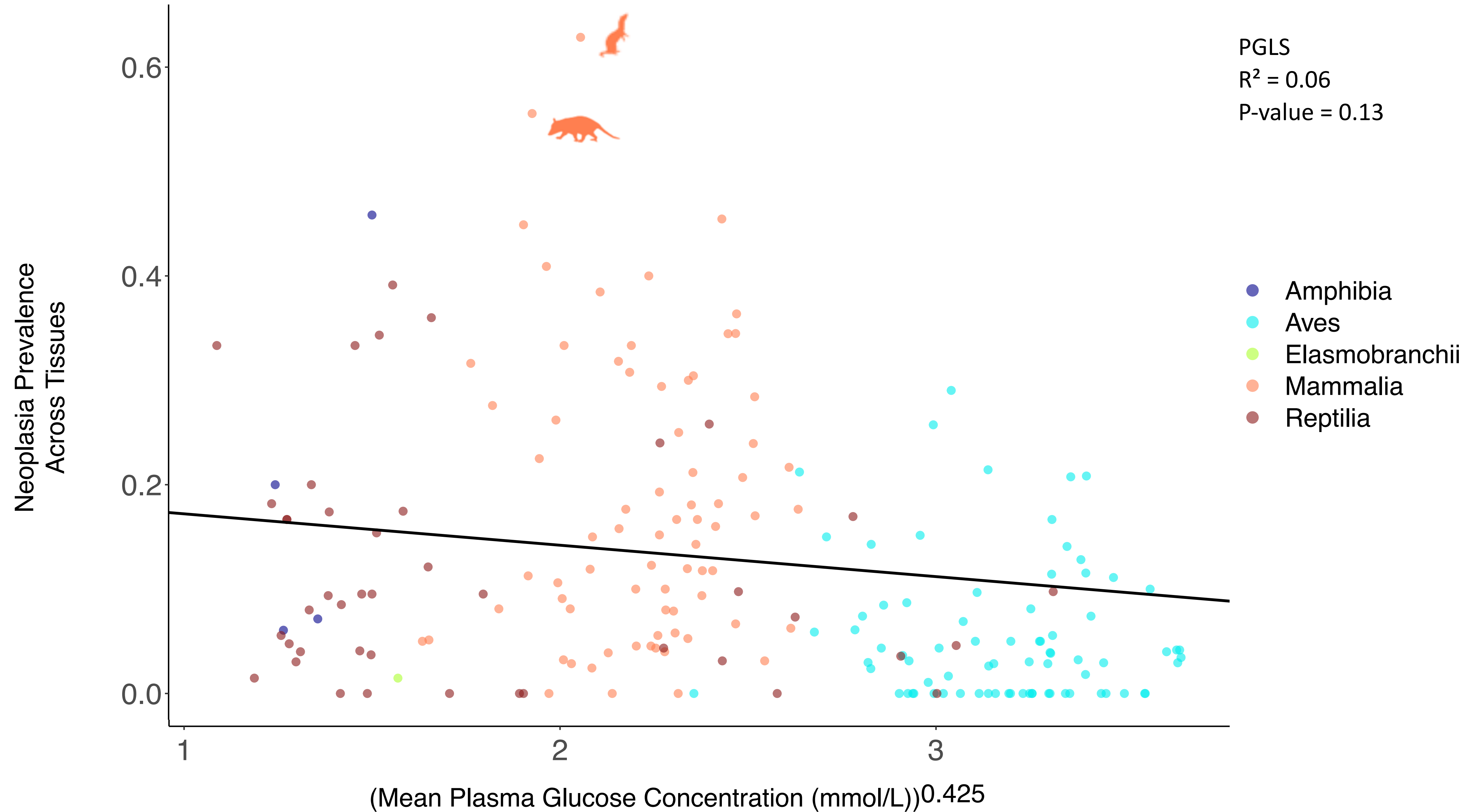

B

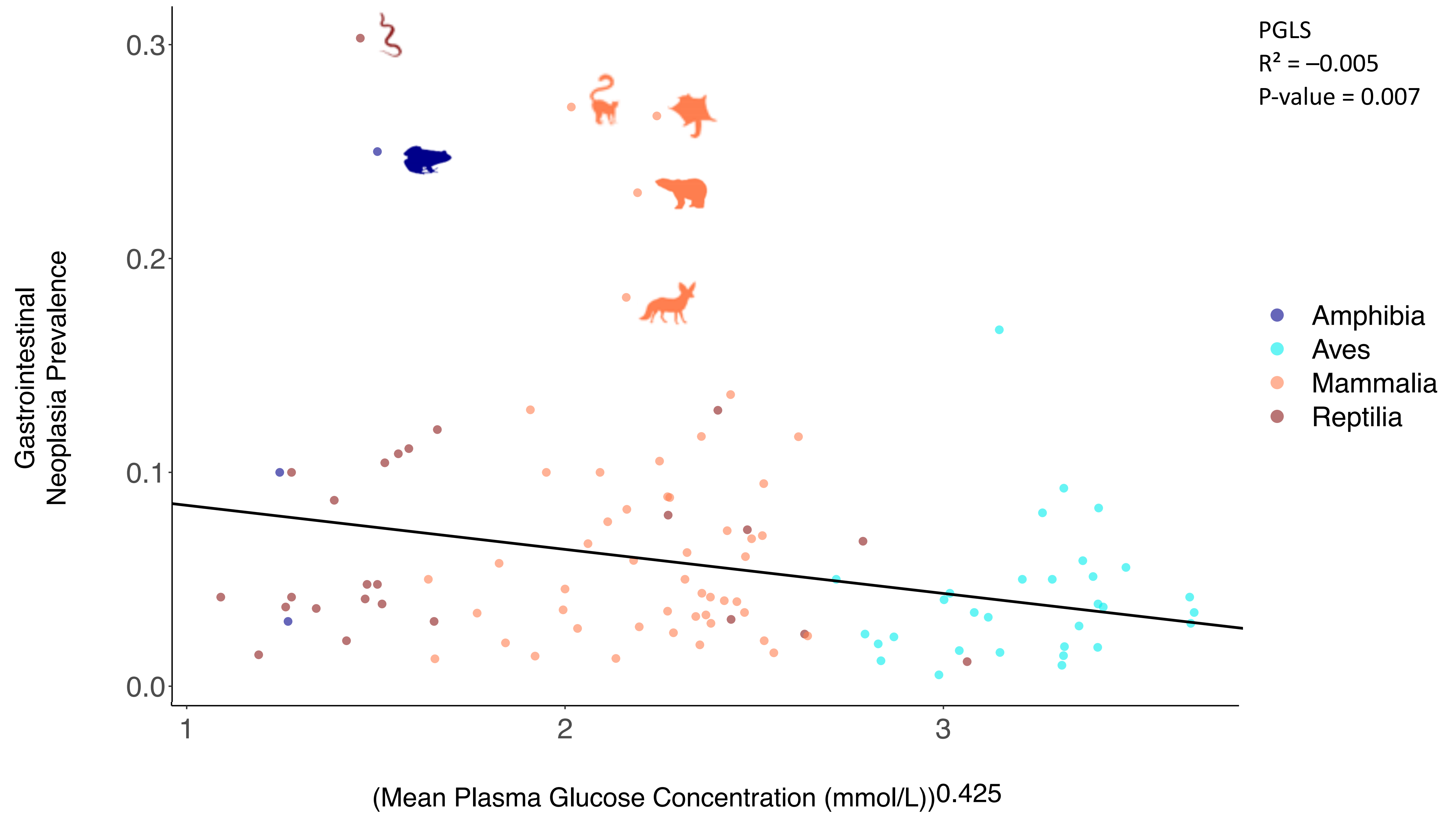

C

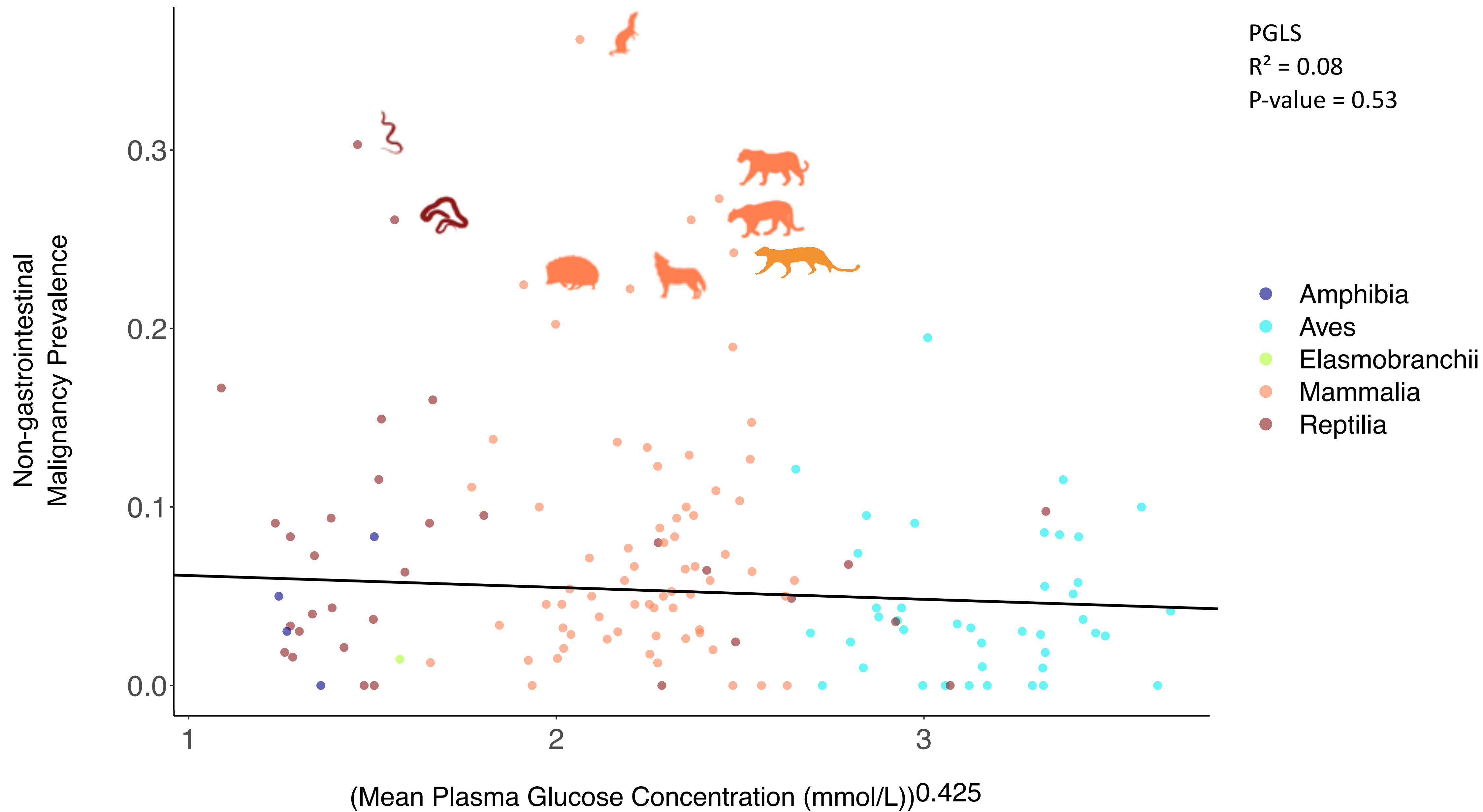

D

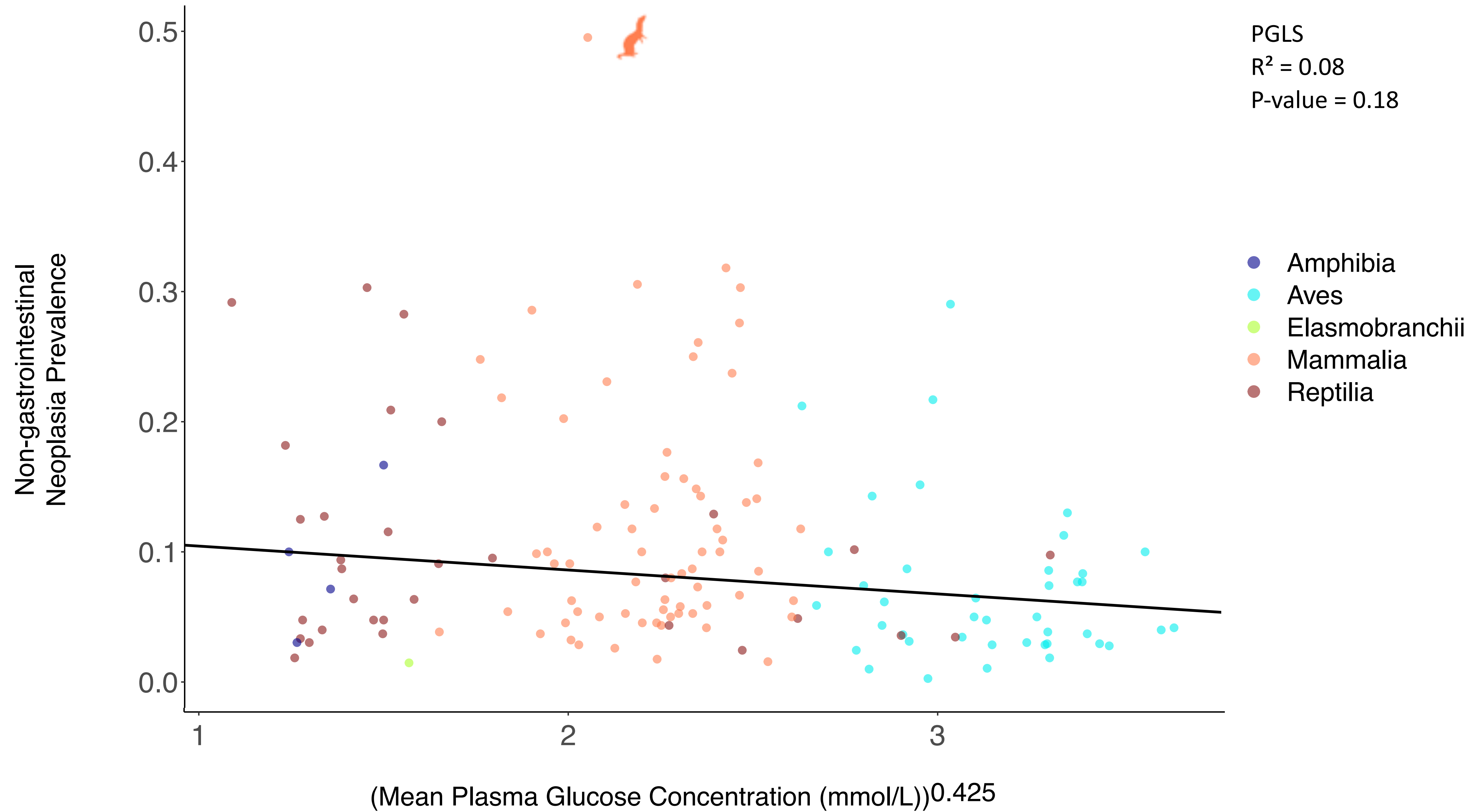

Supplement: Supplement 1 — Supplementary Figure 1. Relationship between malignancy prevalence or neoplasia prevalence and mean plasma glucose concentration. (A) Mean plasma glucose concentration is not significantly correlated with overall neoplasia prevalence for 160 species (PGLS: P-value = 0.13). (B) Mean plasma glucose concentration is negatively correlated with gastrointestinal neoplasia prevalence for 108 species (PGLS: P-value = 0.007) even after correcting for multiple testing (Table 1B). (C) Mean plasma glucose concentration is not significantly correlated with non-gastrointestinal malignancy prevalence for 146 species (PGLS: P-value = 0.53), nor (D) non-gastrointestinal neoplasia prevalence for 146 species (PGLS: P-value = 0.18). Each dot represents the neoplasia prevalence across tissues (A), the gastrointestinal neoplasia prevalence (B), the non-gastrointestinal malignancy prevalence (C), the non-gastrointestinal neoplasia prevalence (D), and the average plasma glucose concentration of one species; Amphibia: Dark Blue; Aves: Blue; Elasmobranchii: Green; Mammalia: Orange; Reptilia: Red. We show images of significant outlier species (Rosner’s test). Animal silhouettes from PhyloPic (http://www.phylopic.org/). [file media-1.pdf]

A

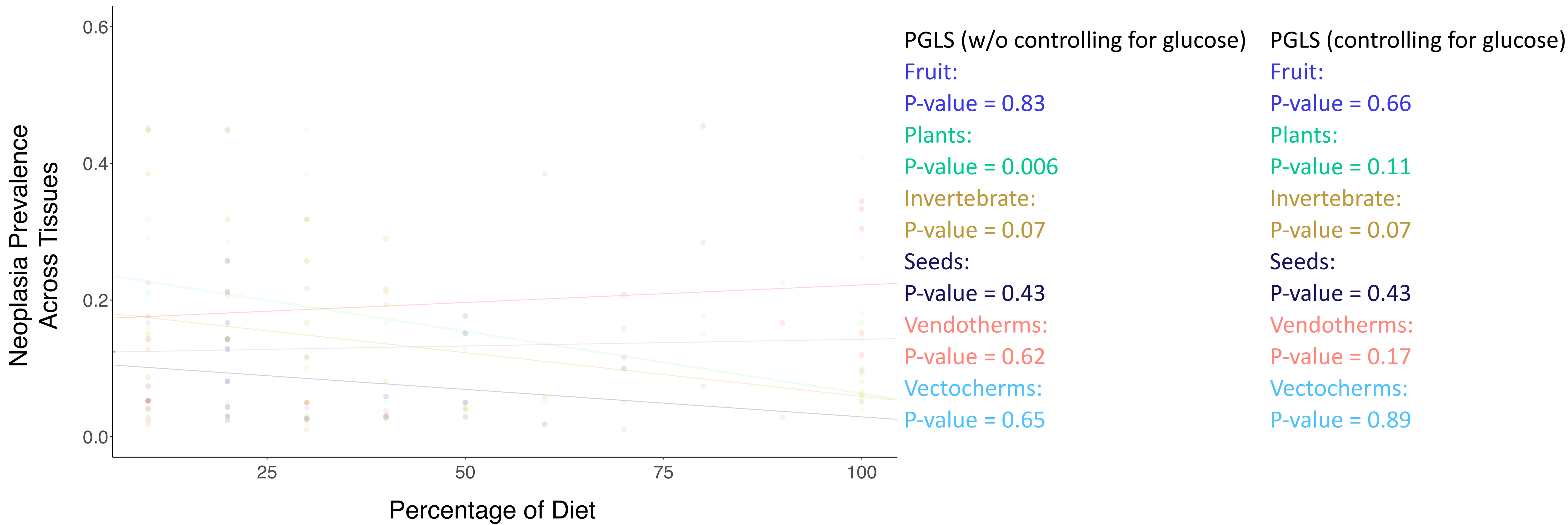

B

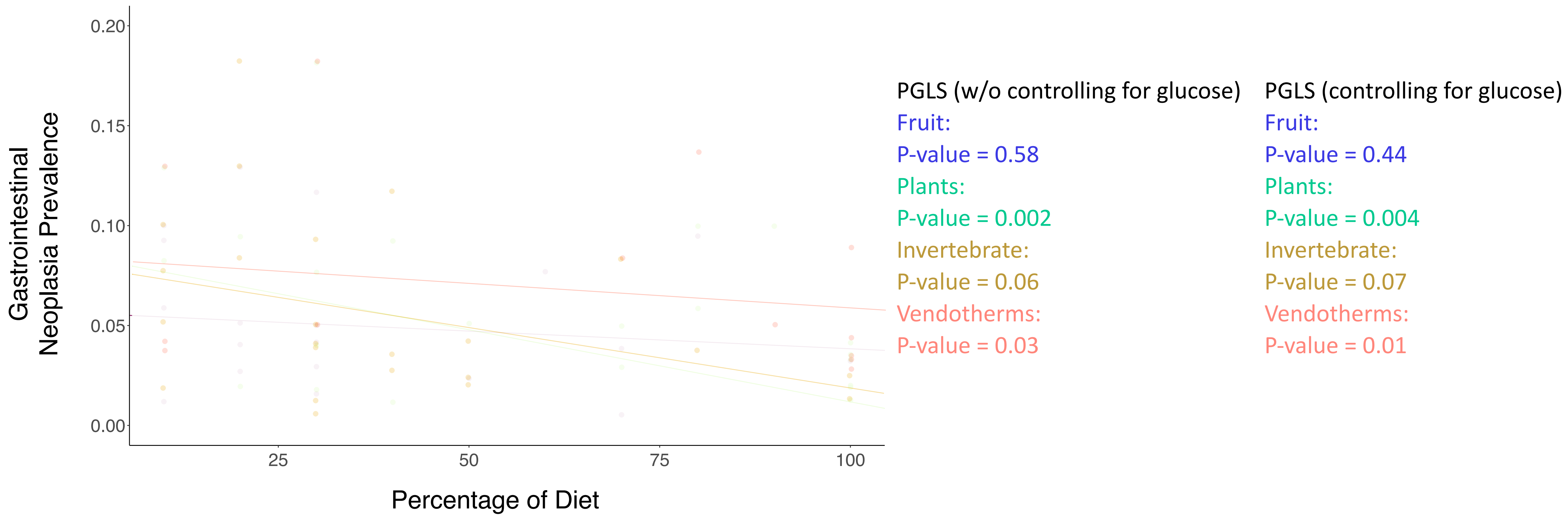

C

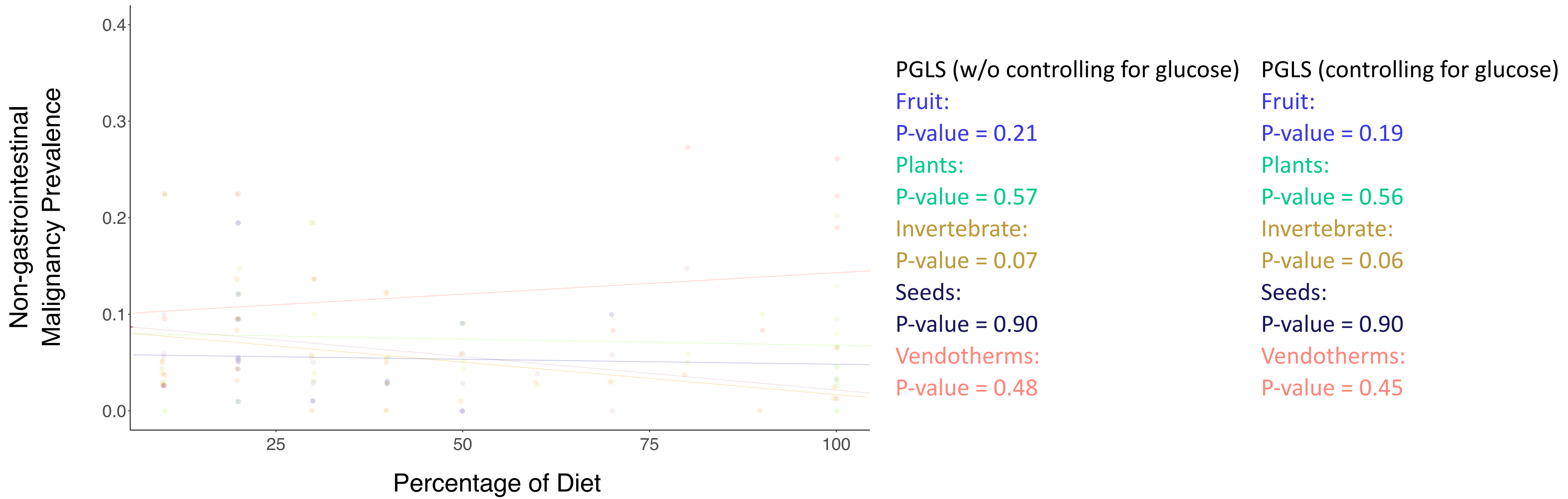

D

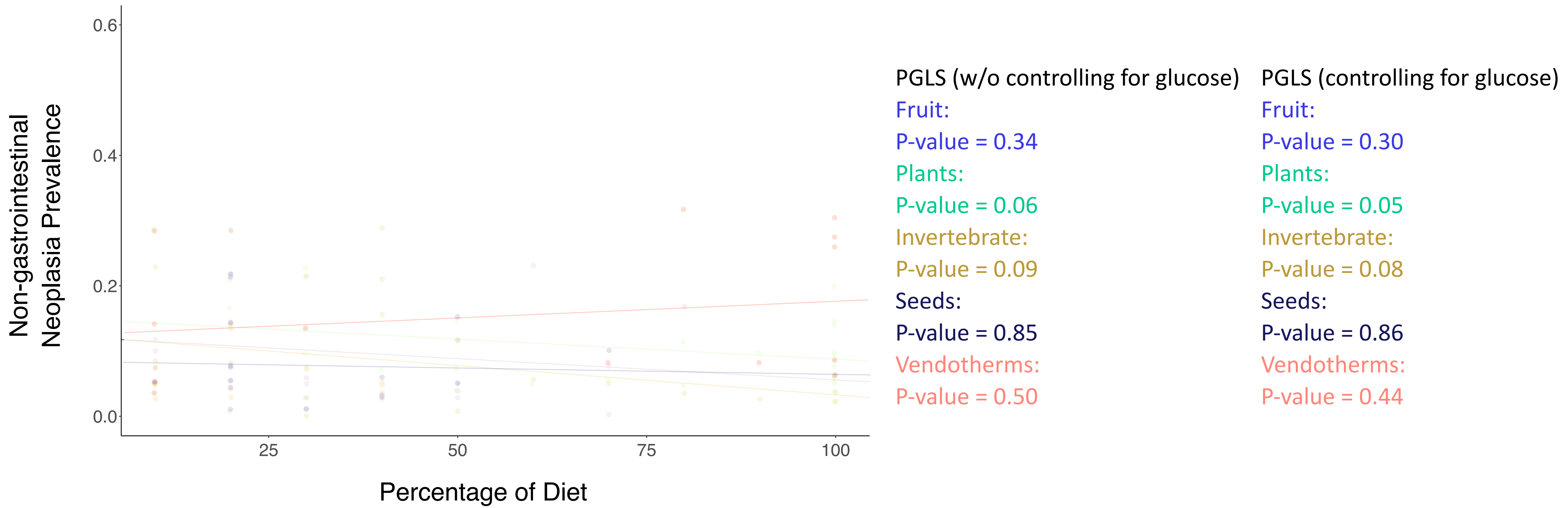

Supplement: Supplement 2 — Supplementary Figure 2. No significant correlation between cancer or neoplasia prevalence and the percentage of food type in a species diet after correcting for multiple testing. (A) The percentage of plants in a species’ diet is negatively correlated with neoplasia prevalence across tissues for 39 species (PGLS: P-value = 0.006), but not when controlling for the variance in their plasma glucose concentrations (PGLS: P-value > 0.05) or after correcting for multiple testing (Table 1F). There is no significant correlation between the percentage of fruit, invertebrates, seeds, endothermic vertebrates (Vendotherms), or ectothermic vertebrates (Vectotherms) in a species’ diet and neoplasia prevalence across tissues for 25, 35, 18, 15 or 11 species, respectively (PGLS: P-value > 0.05). (B) The percentage of plants in a species’ diet is negatively correlated with gastrointestinal neoplasia prevalence for 22 species (PGLS: P-value < 0.05) after correcting for multiple testing, but not when controlling for the variance in their plasma glucose concentrations (Table 1F). The percentage of endothermic vertebrates (Vendotherms) in a species’ diet is negatively correlated with gastrointestinal neoplasia prevalence for 13 species (PGLS: P-value < 0.05), but not after correcting for multiple testing (Table 1F). There is no significant correlation between the percentage of fruit and invertebrates in a species’ diet and gastrointestinal neoplasia prevalence for 19 and 23 species, respectively (PGLS: P-value > 0.05). (C) There is no significant correlation between the percentage of fruit, plants, invertebrates, seeds, or endothermic vertebrates (Vendotherms) in a species’ diet and non-gastrointestinal malignancy prevalence for 22, 36, 31, 16, or 13 species, respectively (PGLS: P-value > 0.05). (D) There is no significant correlation between the percentage of fruit, plants, invertebrates, seeds, or endothermic vertebrates (Vendotherms) in a species’ diet and non-gastrointestinal neopla [file media-2.pdf]

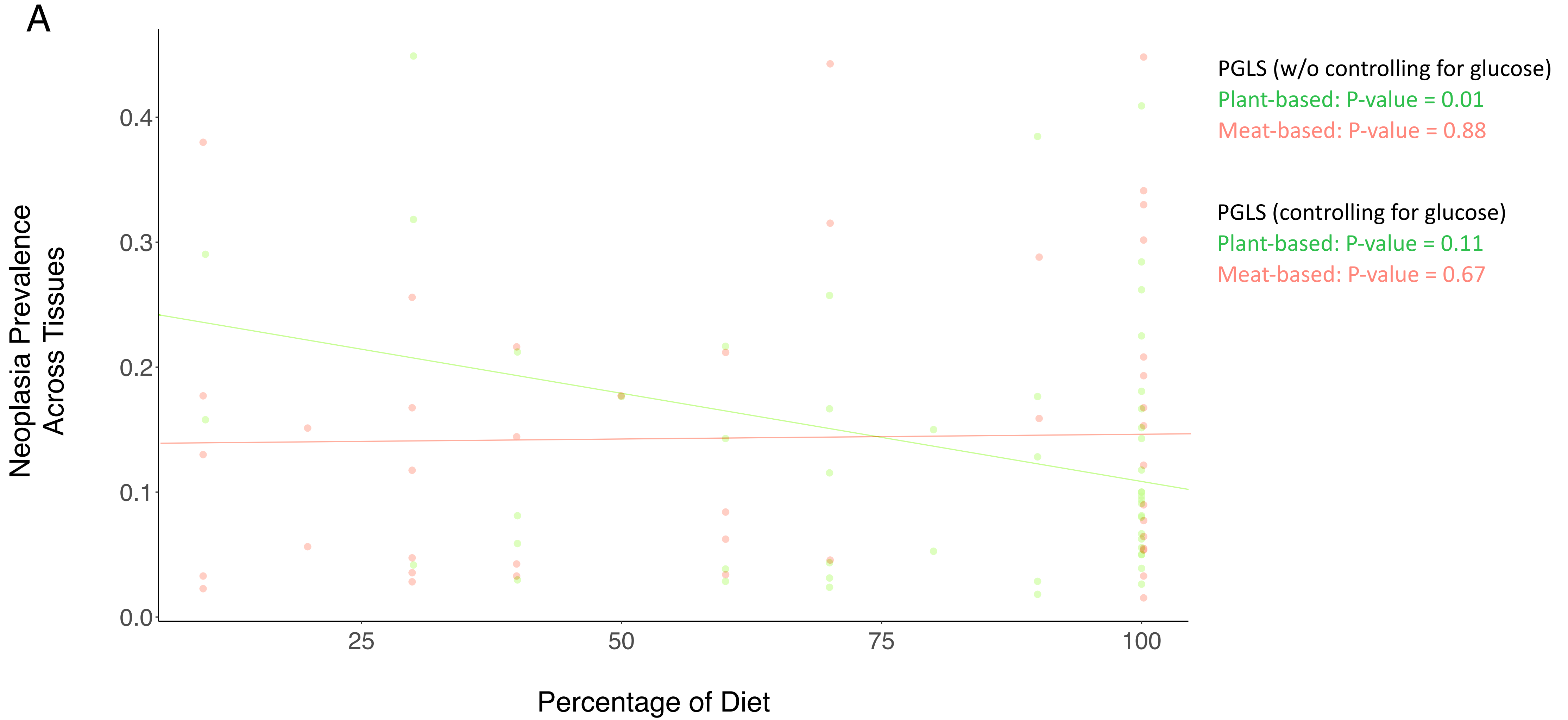

**B**

Gastrointestinal  
Neoplasia Prevalence

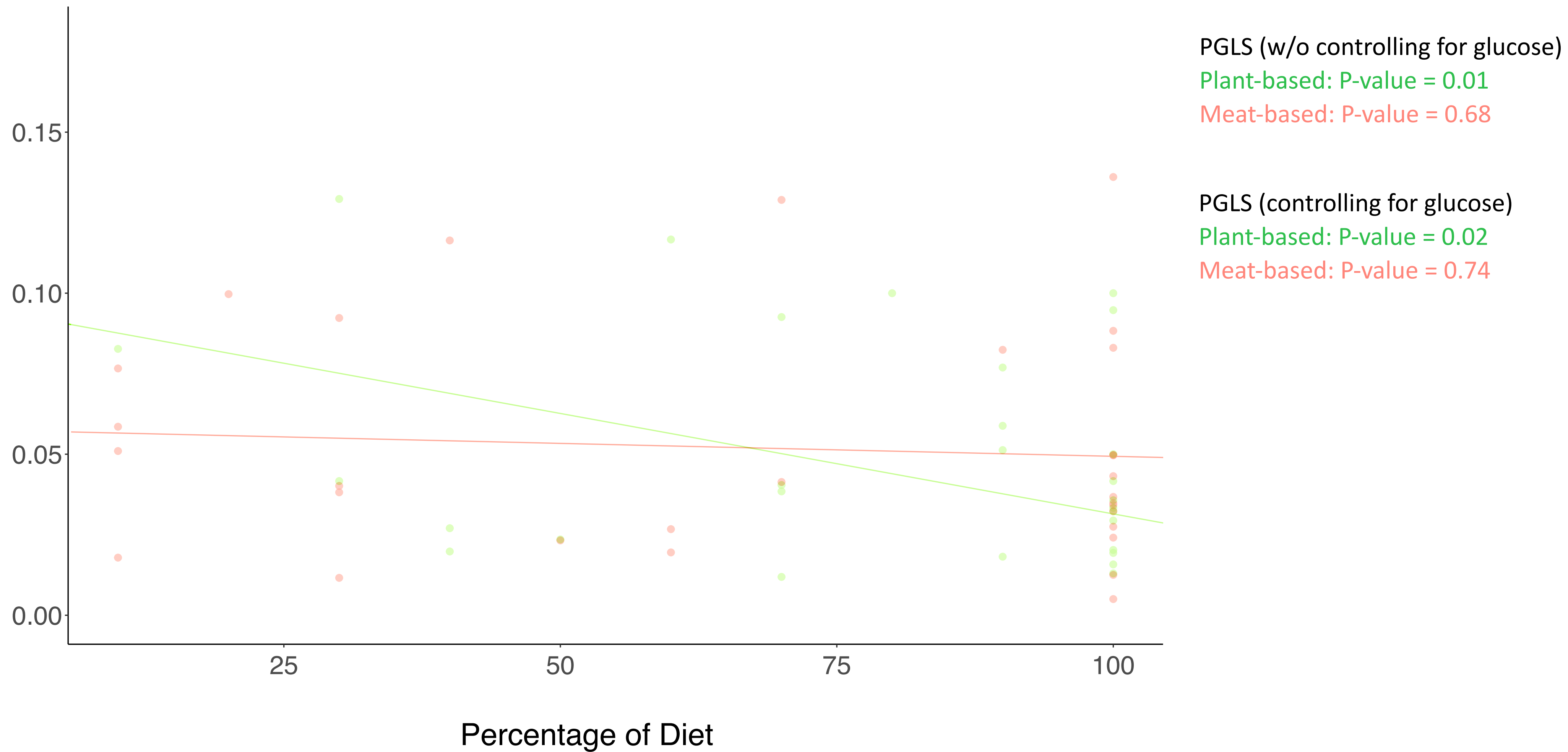

C

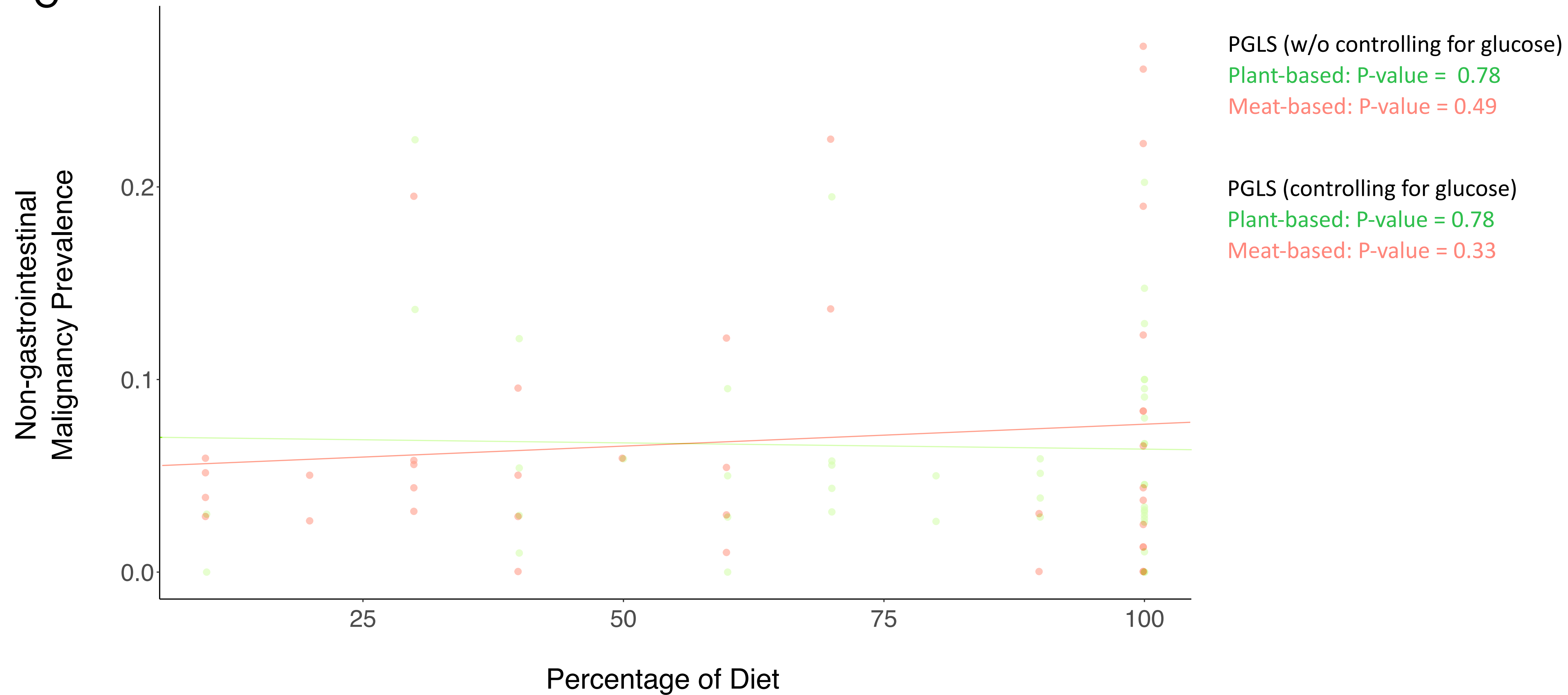

D

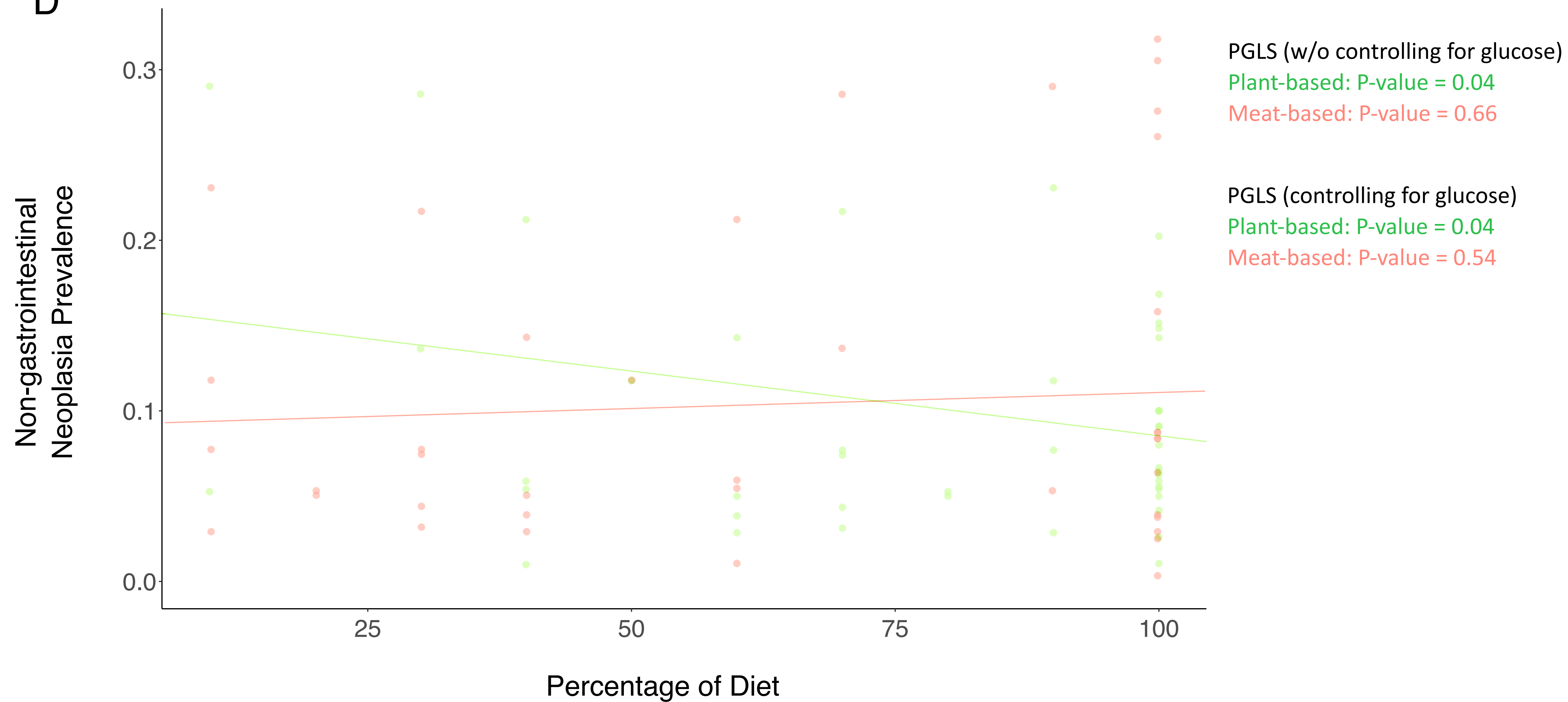

Supplement: Supplement 3 — Supplementary Figure 3. No significant correlation between malignancy prevalence or neoplasia prevalence and percentage of plant-based or meat-based food in species’ diet after correcting for multiple testing. (A) The percentage of plant-based food in a species’ diet is negatively correlated with neoplasia prevalence across tissues for 50 species without controlling for the variance in their plasma glucose concentrations (PGLS: P-value = 0.01), but not after correcting for multiple testing (Table 1F). There is no significant correlation between the percentage of animal-based food in a species’ diet and neoplasia prevalence across tissues for 44 species (PGLS: P-value > 0.05). (B) The percentage of plant-based food in a species’ diet is negatively correlated with gastrointestinal neoplasia prevalence for 29 species with (PGLS: P-value = 0.02) or without (PGLS: P-value = 0.01) controlling for the variance in their plasma glucose concentrations, but not after applying corrections for multiple testing (Table 1F). There is no significant correlation between the percentage of animal-based food in a species’ diet and gastrointestinal neoplasia prevalence for 32 species (PGLS: P-value > 0.05). (C) The percentage of plant-based or animal-based food in a species’ diet is not significantly correlated with non-gastrointestinal malignancy prevalence for 46 or 39 species, respectively (PGLS: P-value > 0.05). (D) The percentage of plant-based or animal-based food in a species’ diet is not significantly correlated with non-gastrointestinal neoplasia prevalence for 46 or 39 species after correcting for multiple testing. Each dot shows the neoplasia prevalence across tissues (A), the gastrointestinal neoplasia prevalence (B), the non-gastrointestinal malignancy prevalence (C), the non-gastrointestinal neoplasia prevalence (D), and the percentage of plant-based or meta-based food in the diet of one species. We added minimal jitter in the plots in order to better visualize individual d [file media-3.pdf]

A

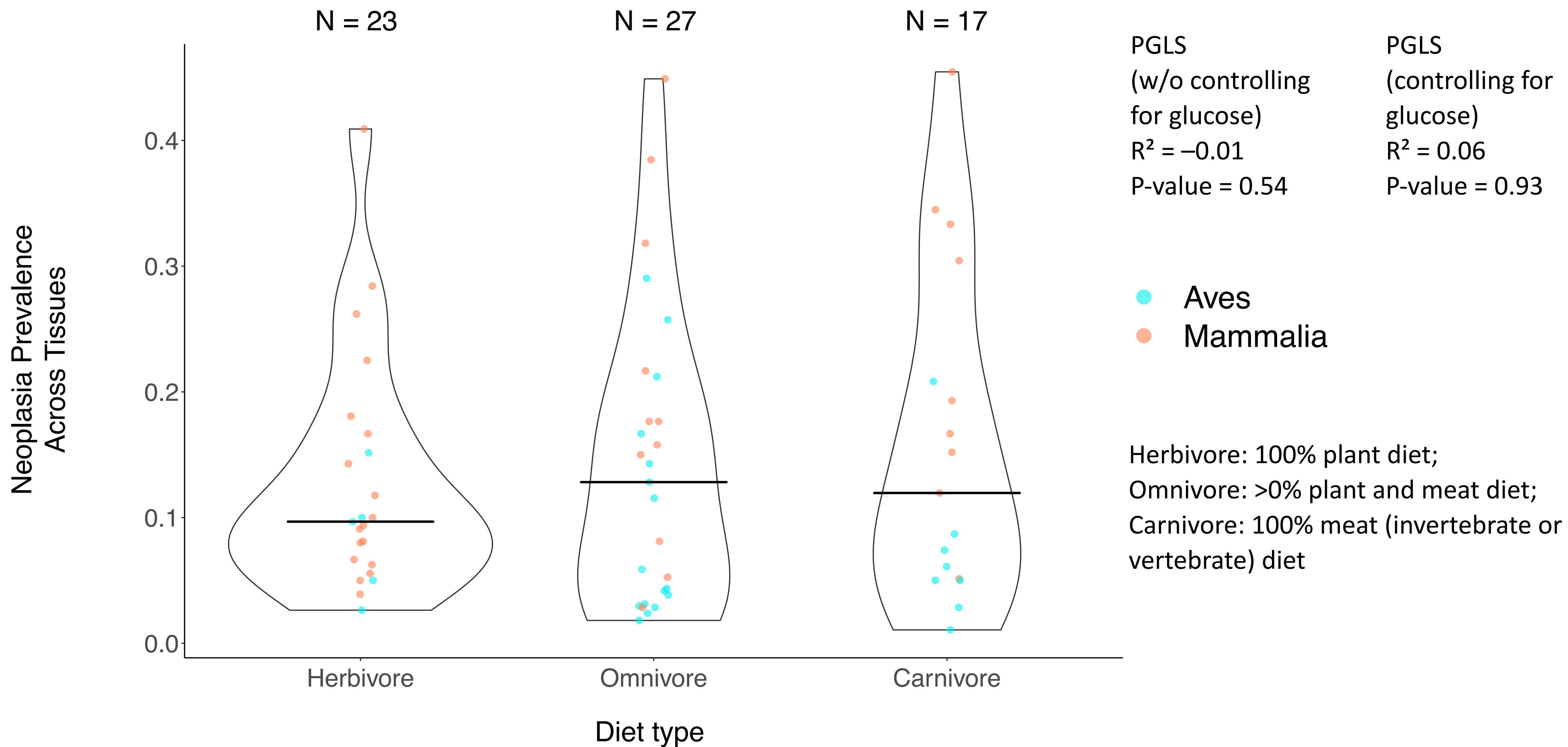

B

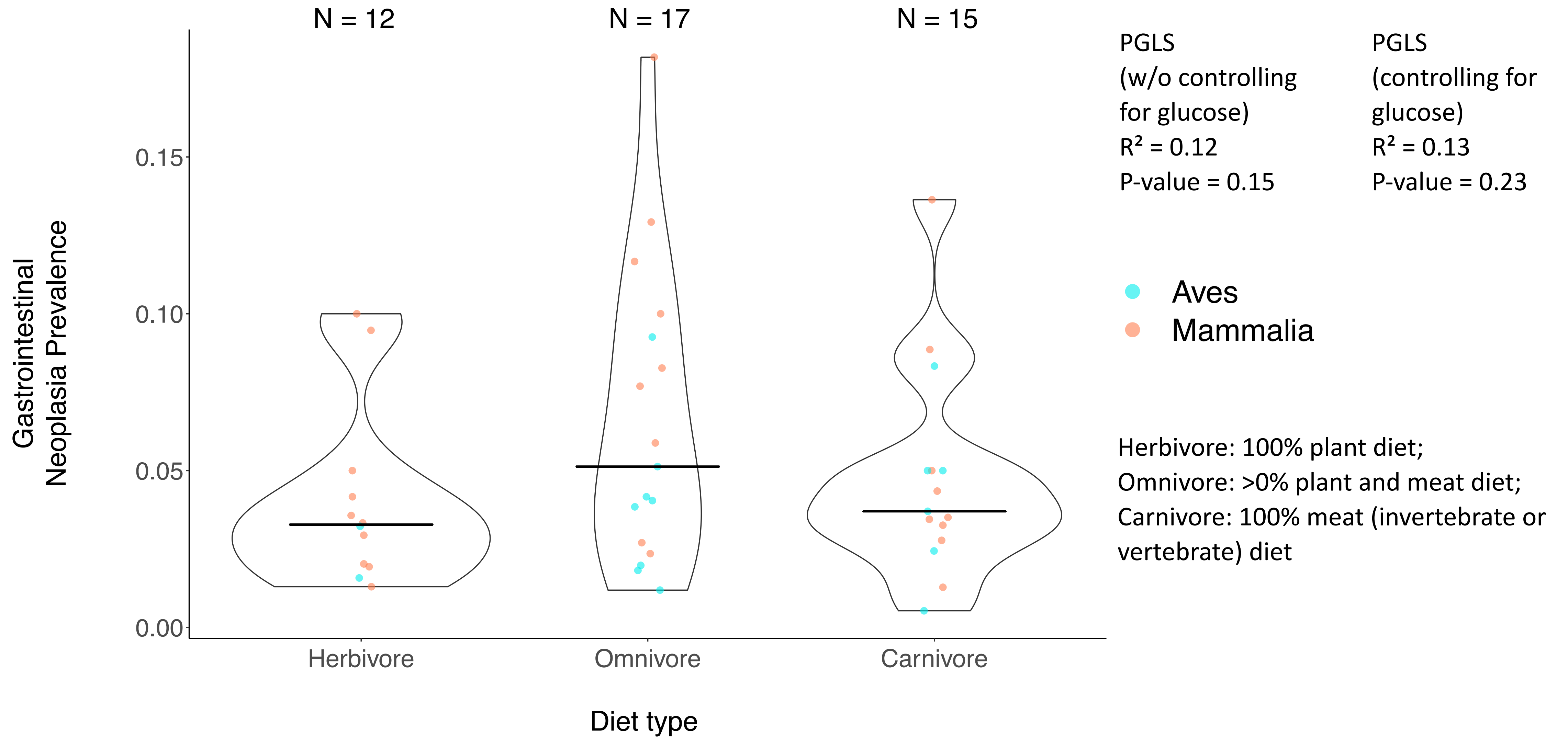

C

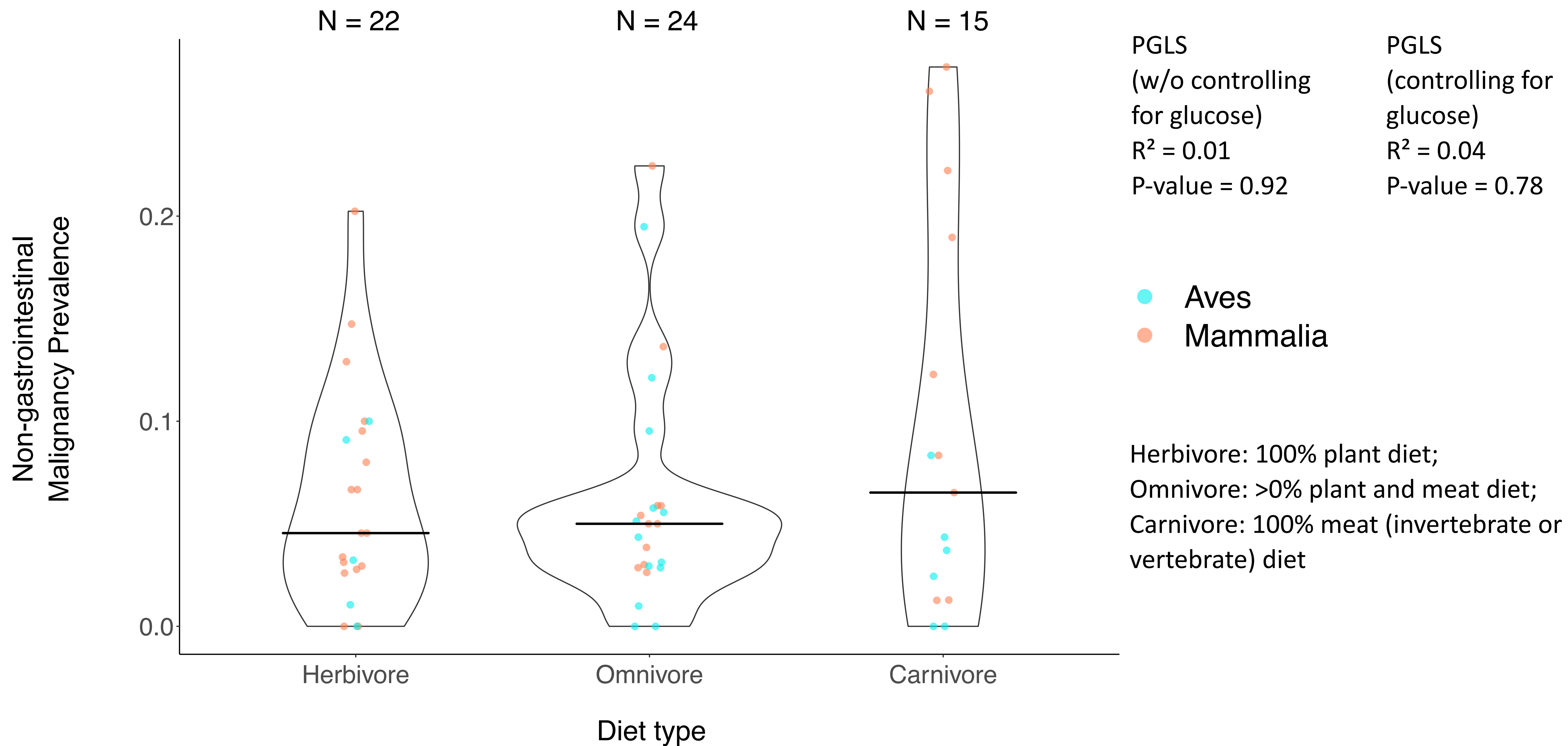

D

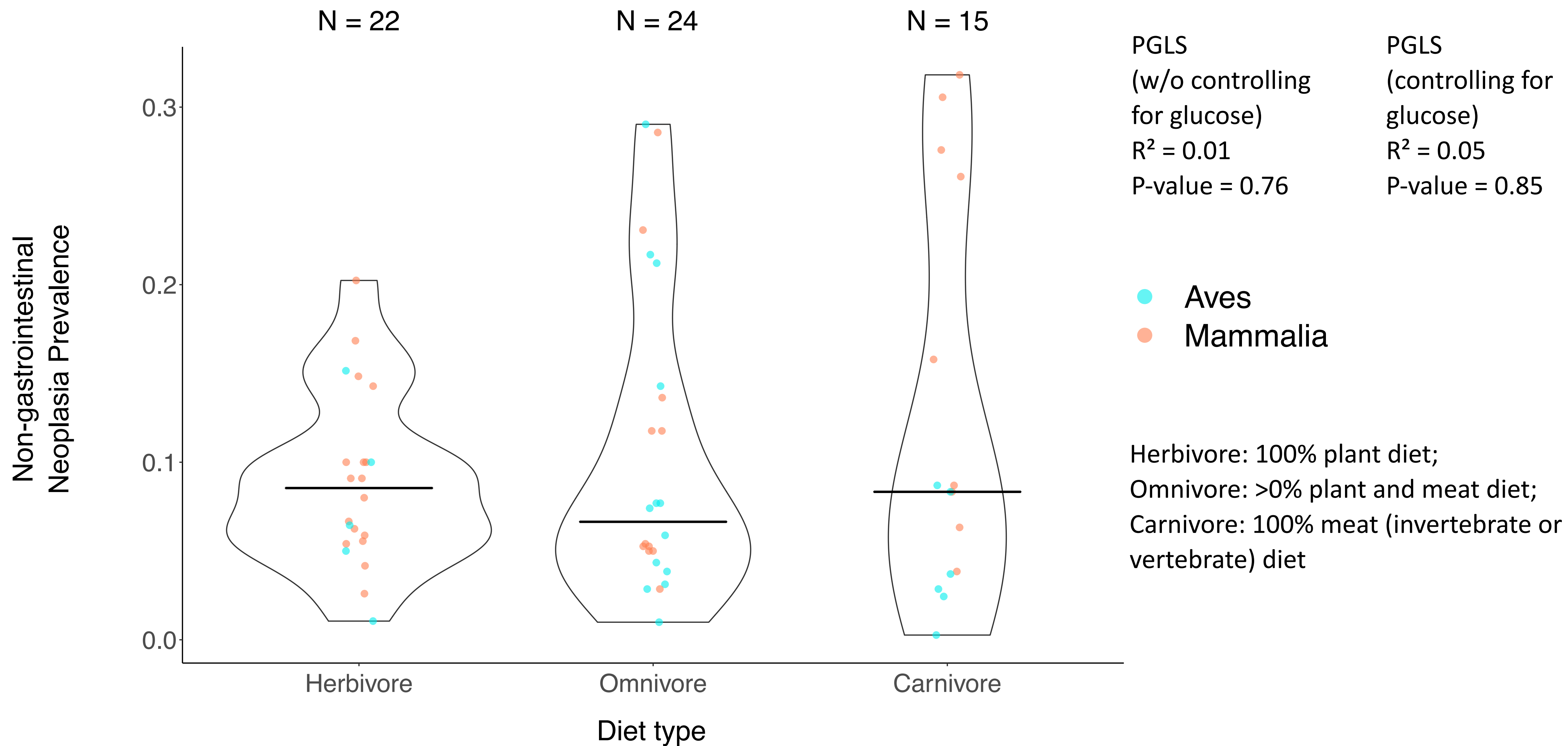

Supplement: Supplement 4 — Supplementary Figure 4. No significant correlation between malignancy prevalence or neoplasia prevalence and diet type. Diet type is not significantly correlated with neoplasia across tissues (A), gastrointestinal neoplasia prevalence (B), non-gastrointestinal malignancy prevalence (C), or non-gastrointestinal neoplasia prevalence (D) (PGLS: P-value > 0.05). Each dot shows the neoplasia prevalence across tissues (A), the gastrointestinal neoplasia prevalence (B), the non-gastrointestinal malignancy prevalence (C), the non-gastrointestinal neoplasia prevalence (D), and the diet type of one species. N shows the number of species per diet category. The horizontal black line in each diet category shows the median neoplasia prevalence across tissues (A), the median gastrointestinal neoplasia prevalence (B), the median non-gastrointestinal malignancy prevalence (C), or the median non-gastrointestinal neoplasia prevalence (D), in that diet category. [file media-4.pdf]

A

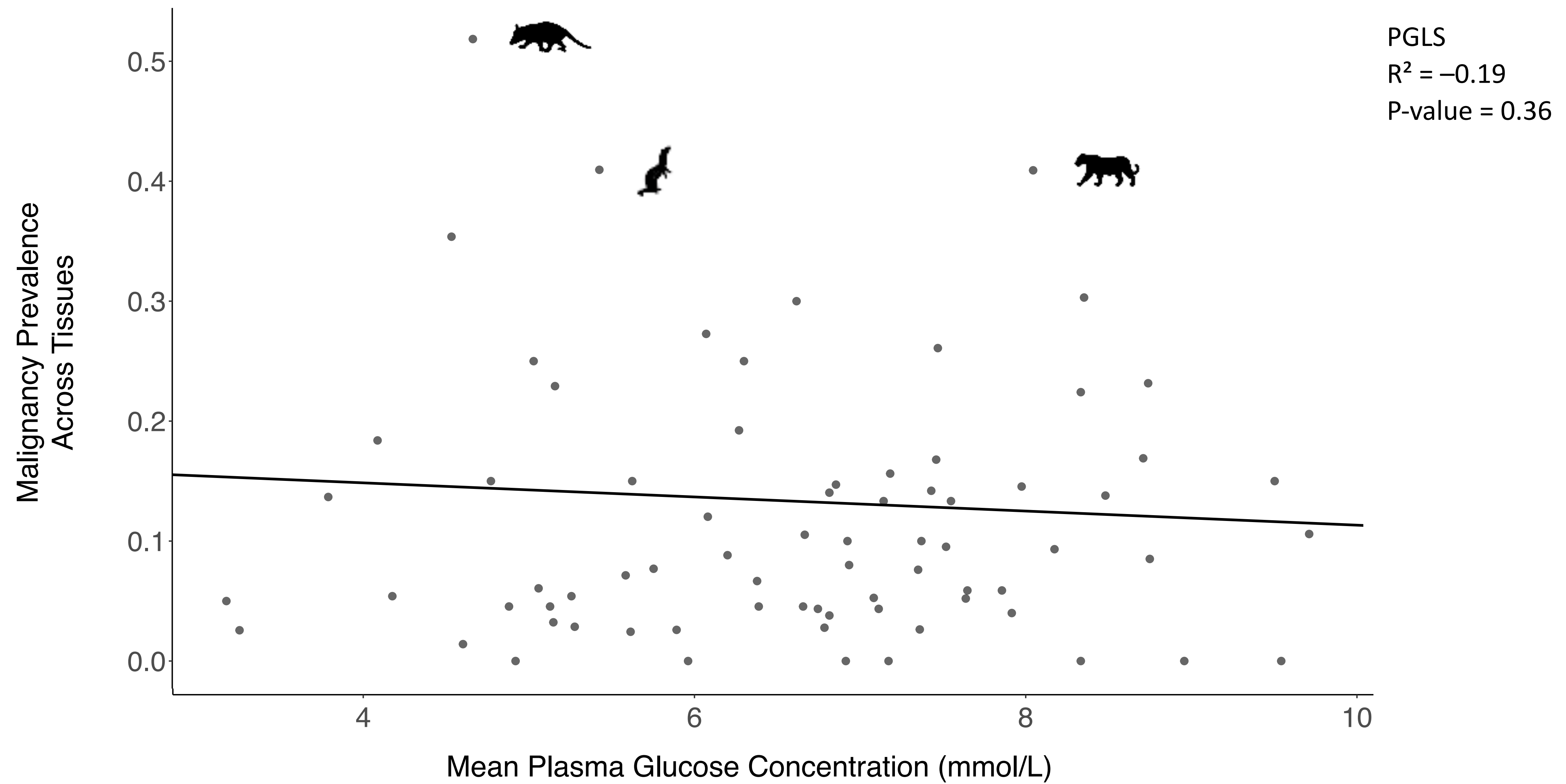

B

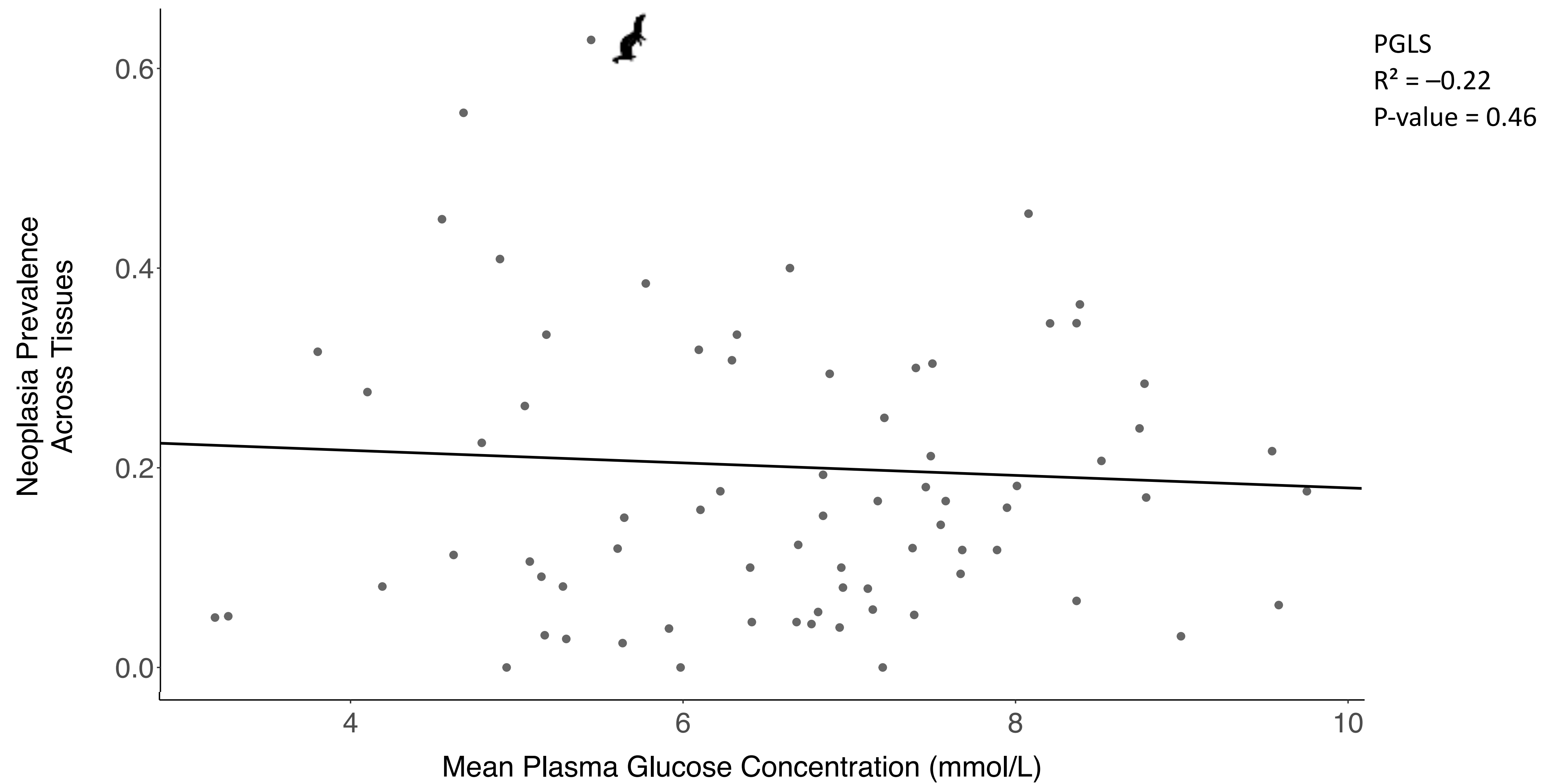

C

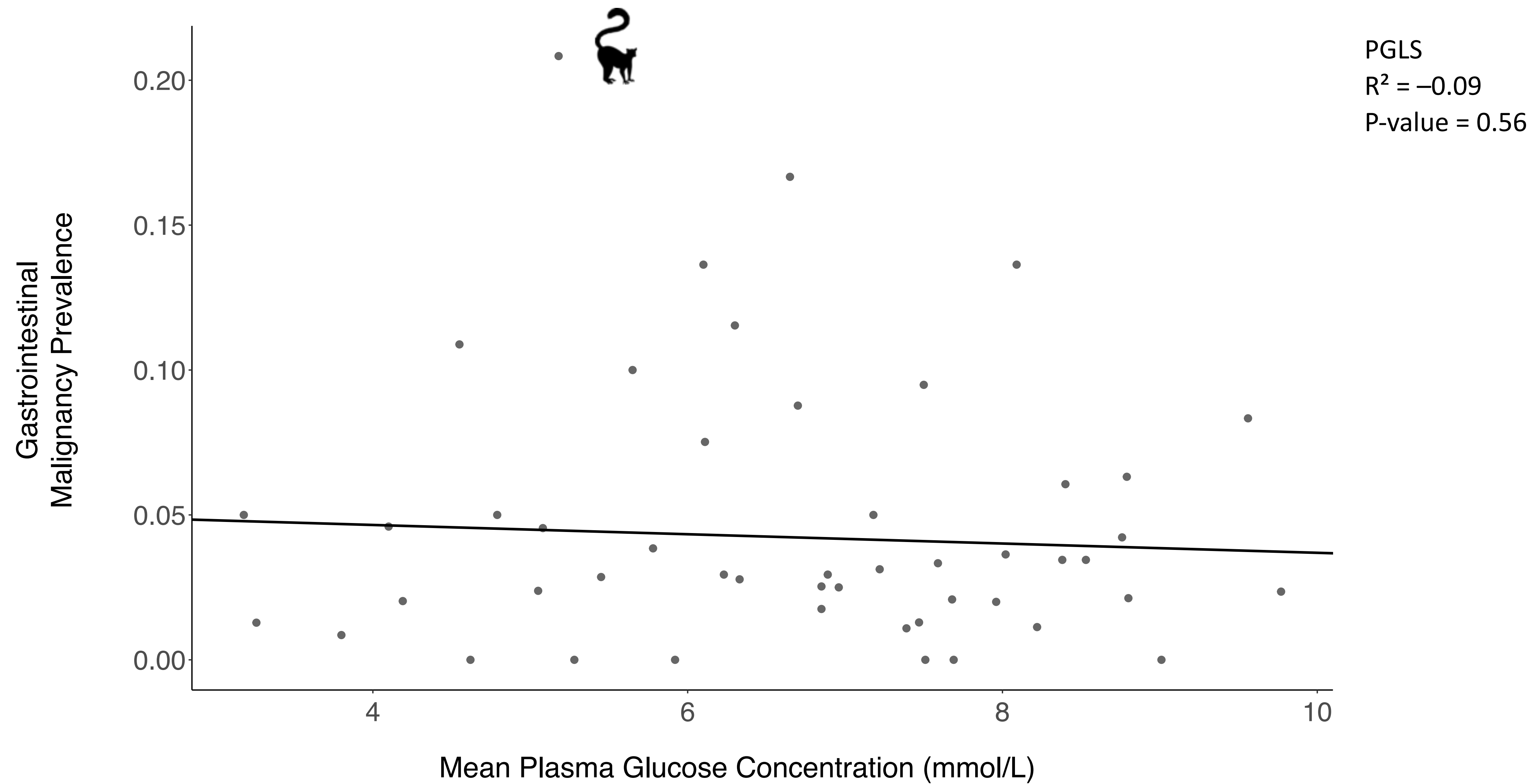

D

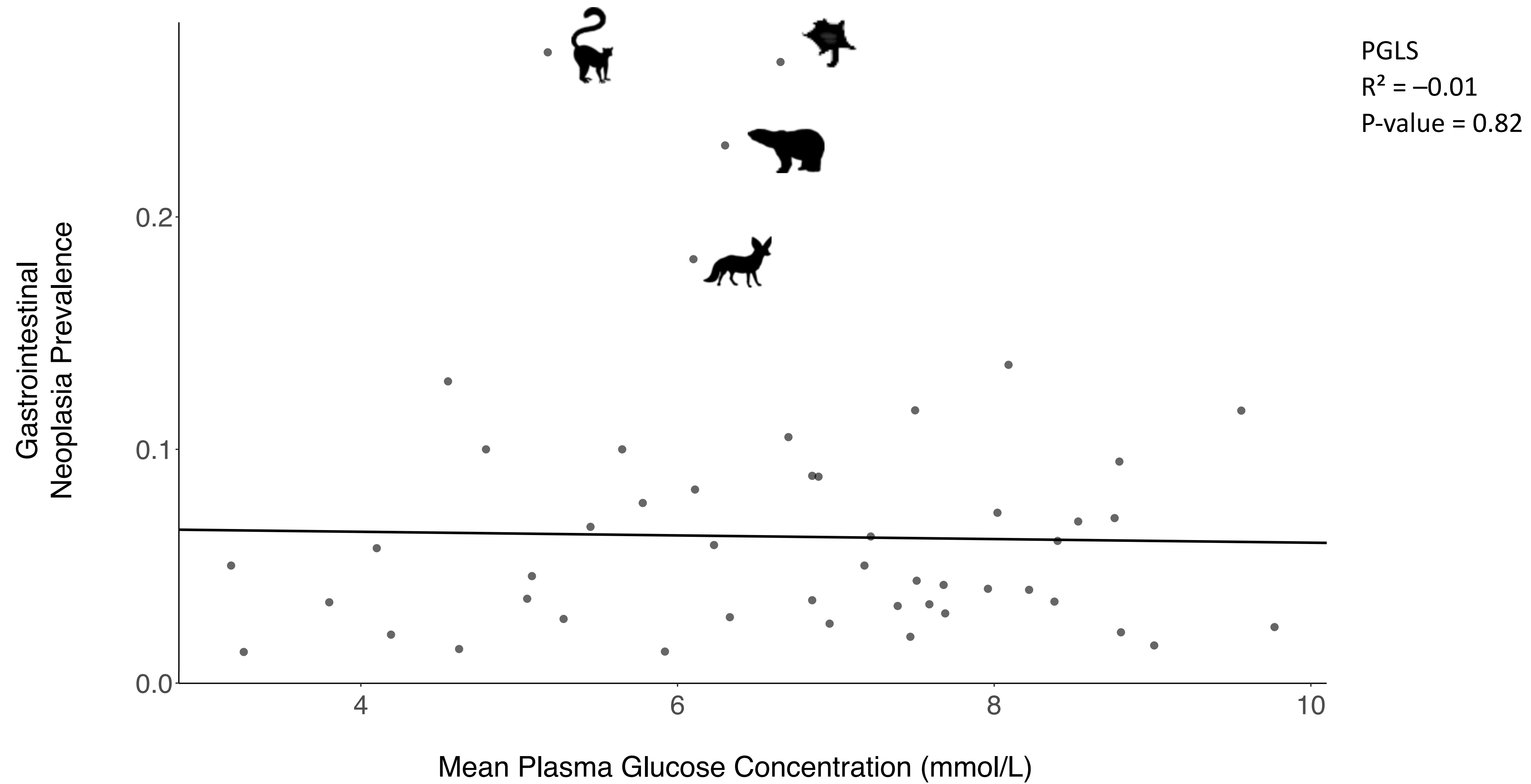

E

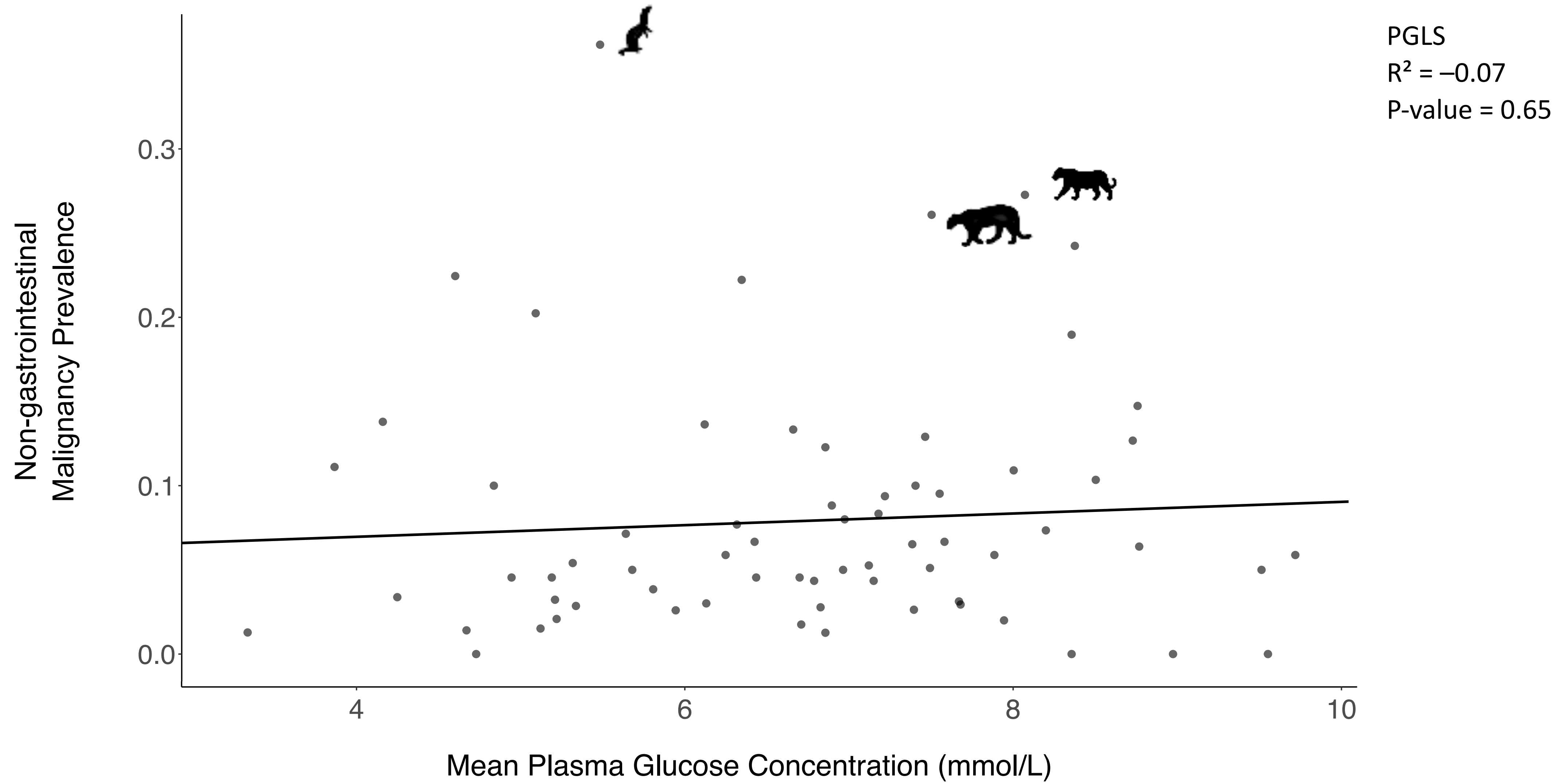

F

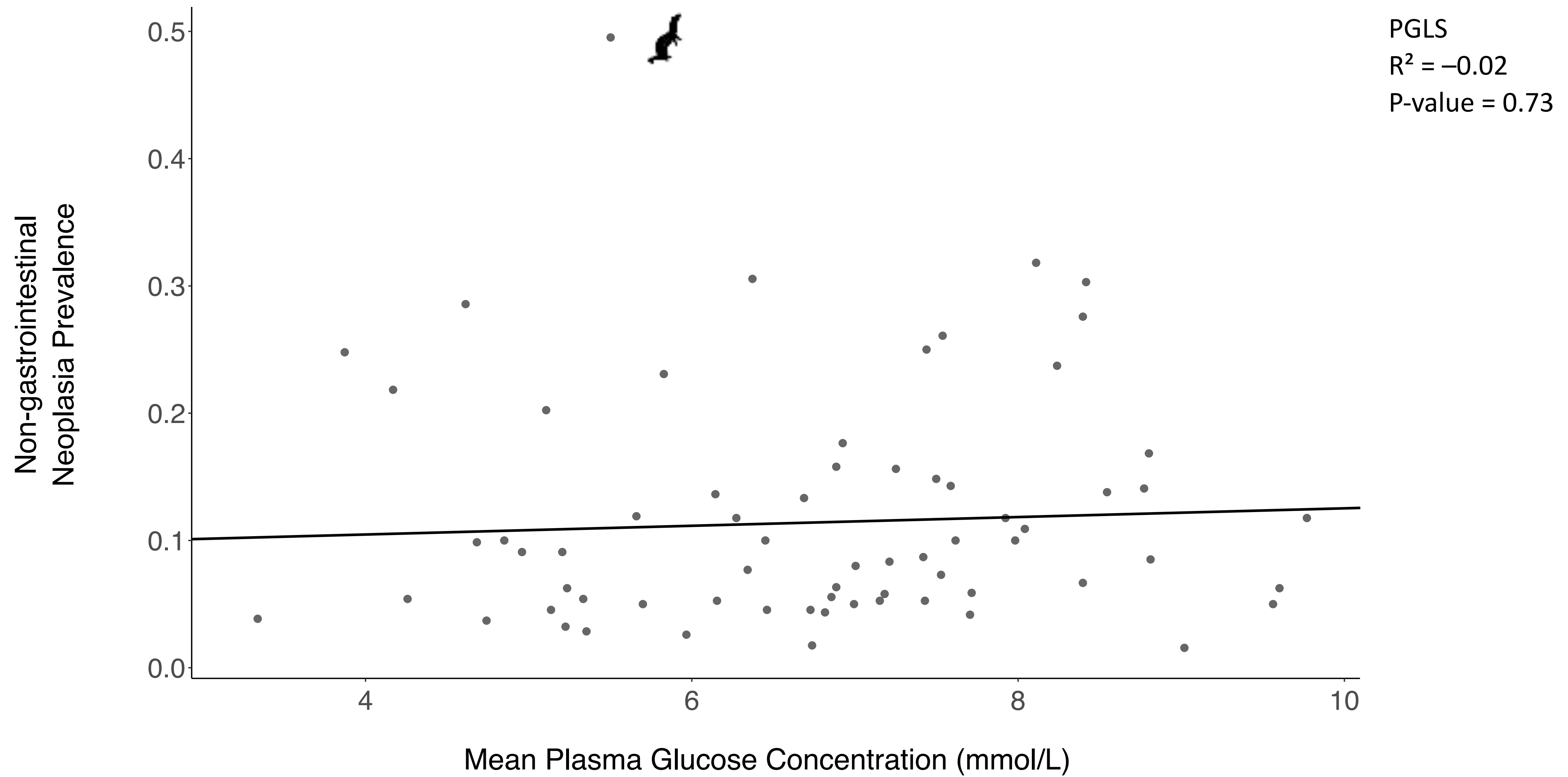

Supplement: Supplement 5 — Supplementary Figure 5. No significant correlation between cancer or neoplasia prevalence and mean plasma glucose concentration in mammals. There is no significant correlation between mean plasma glucose concentration and (A) malignancy prevalence across tissues for 68 mammalian species, (B) neoplasia prevalence across tissues for 68 mammalian species, (C) gastrointestinal malignancy prevalence for 48 mammalian species, (D) gastrointestinal neoplasia prevalence for 48 mammalian species, (E) non-gastrointestinal malignancy prevalence for 67 mammalian species, and (F) non-gastrointestinal neoplasia prevalence for 67 mammalian species (PGLS: P-value > 0.05). Each dot shows the malignancy prevalence across tissues (A), the neoplasia prevalence across tissues (B), the gastrointestinal malignancy prevalence (C), the gastrointestinal neoplasia prevalence (D), the non-gastrointestinal malignancy prevalence (E), the non-gastrointestinal neoplasia prevalence (F), and the average plasma glucose concentration of one species. We show images of significant outlier species (Rosner’s test). Animal silhouettes from PhyloPic (http://www.phylopic.org/). [file media-5.pdf]

A

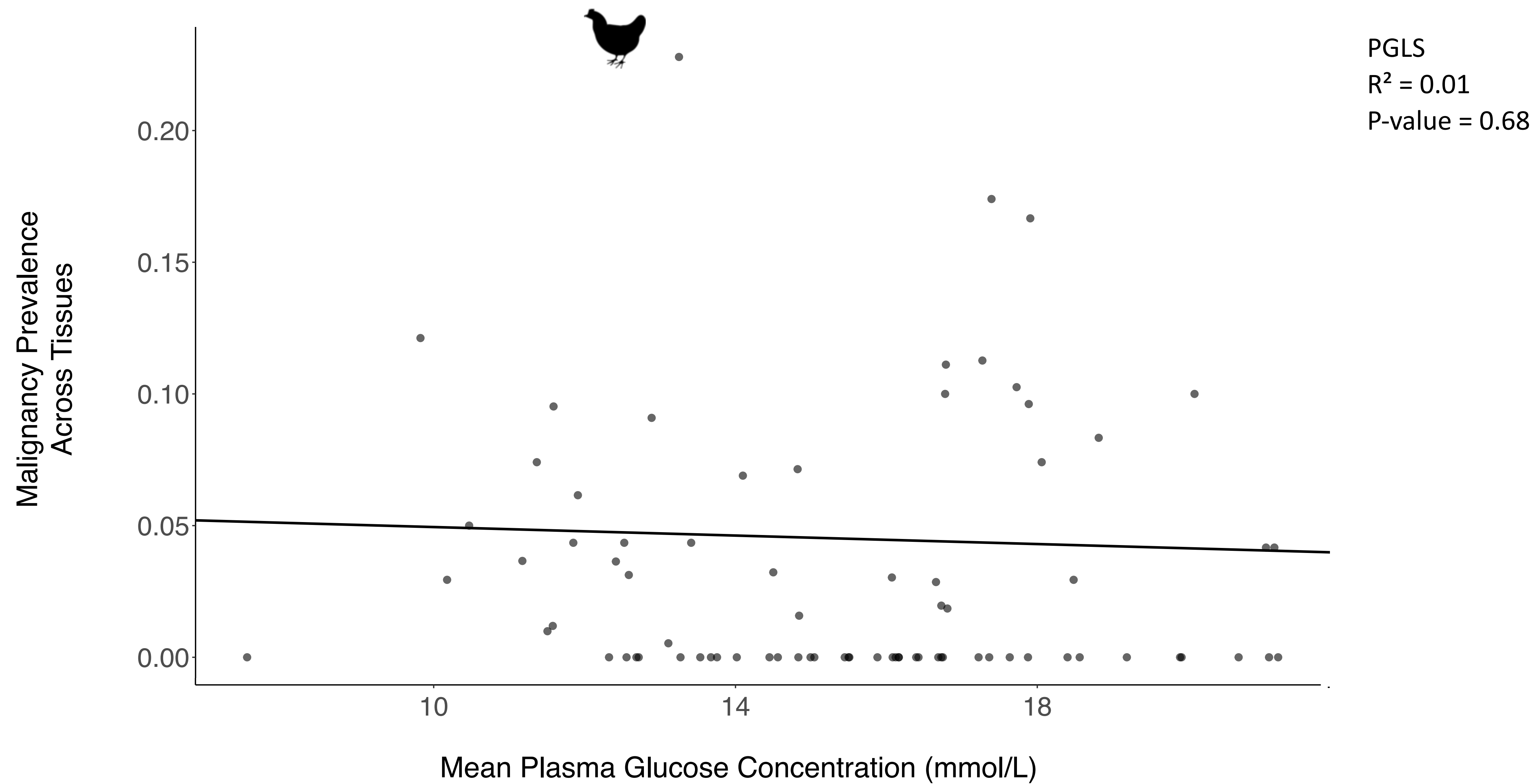

B

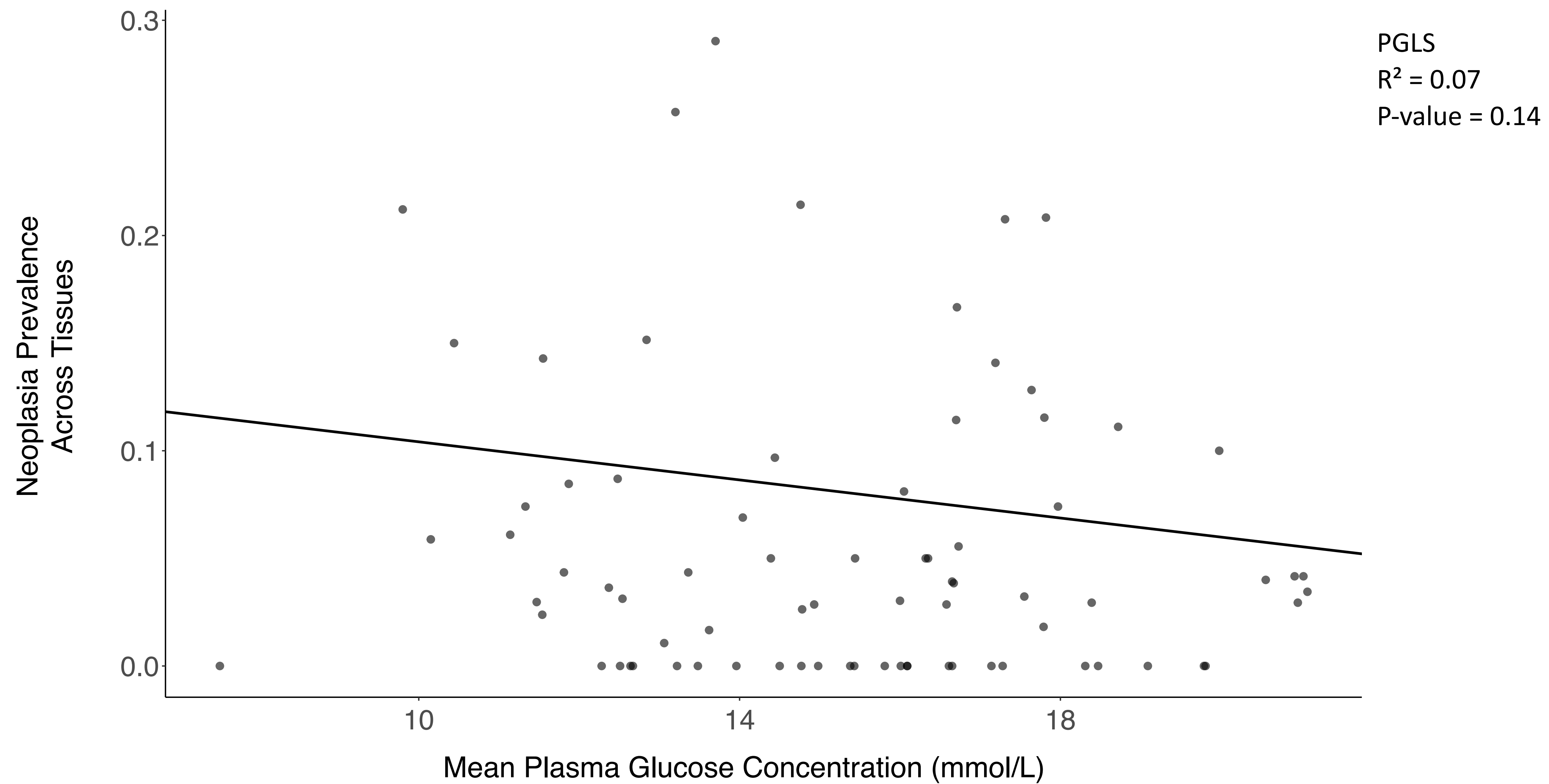

C

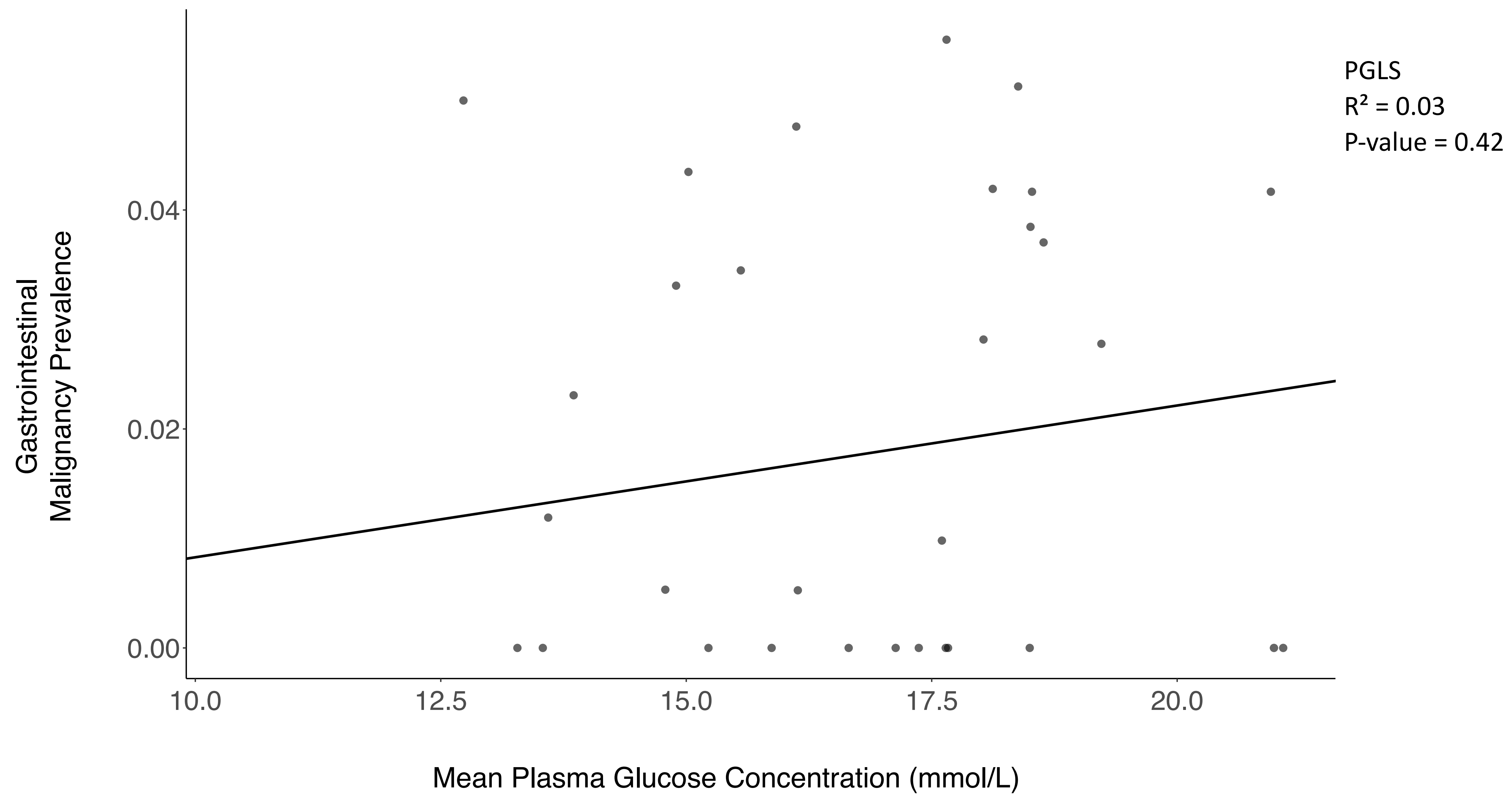

D

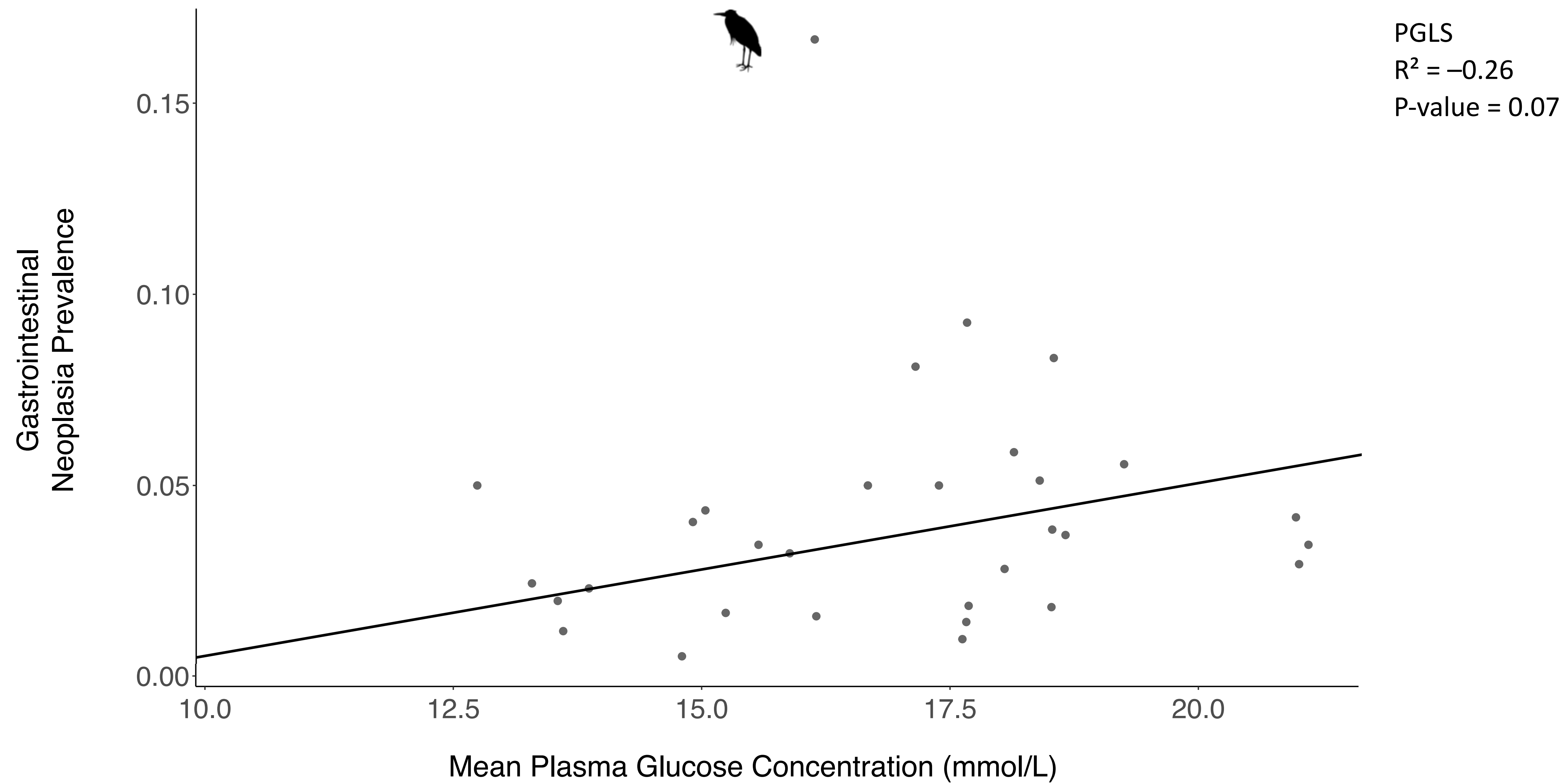

E

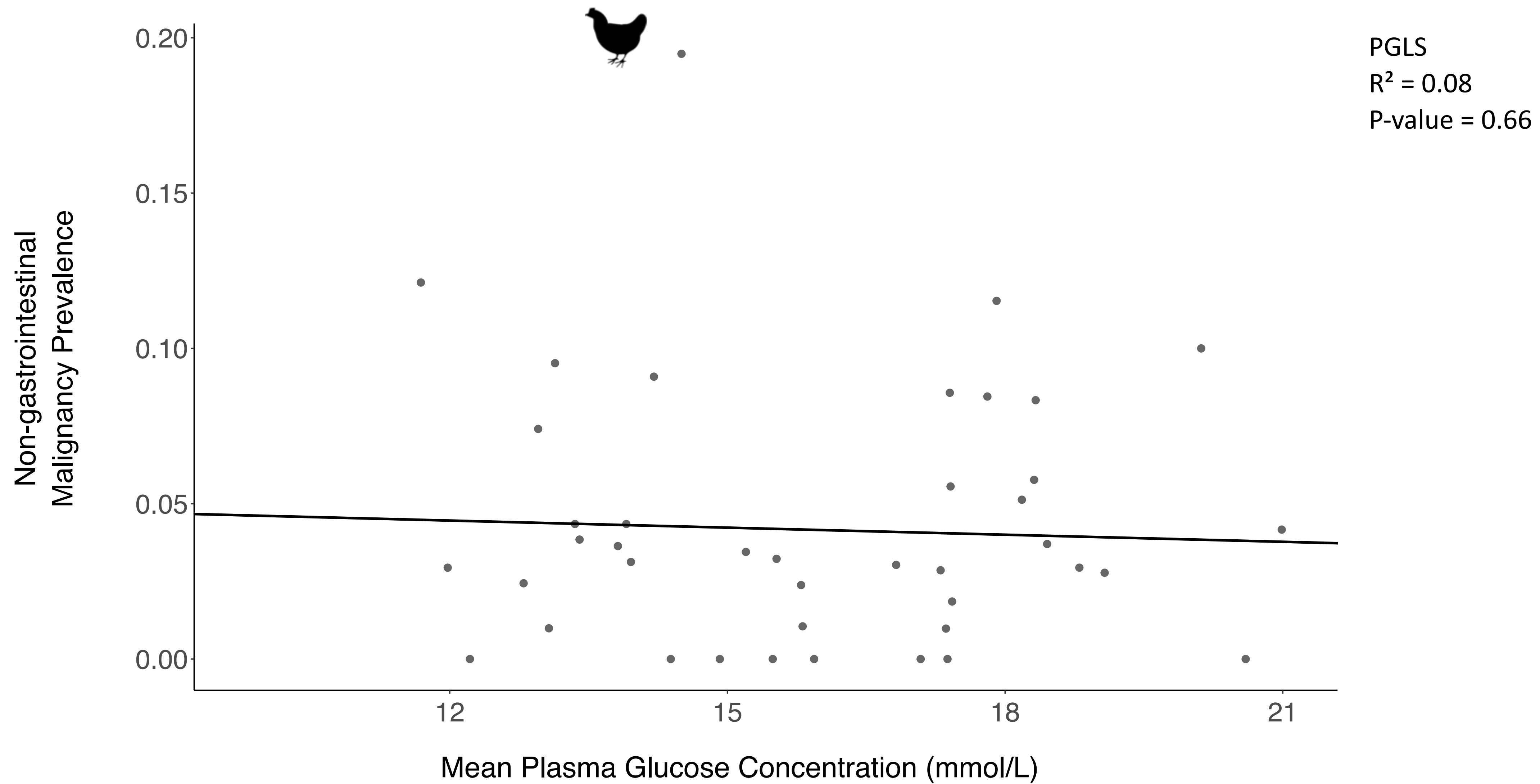

F

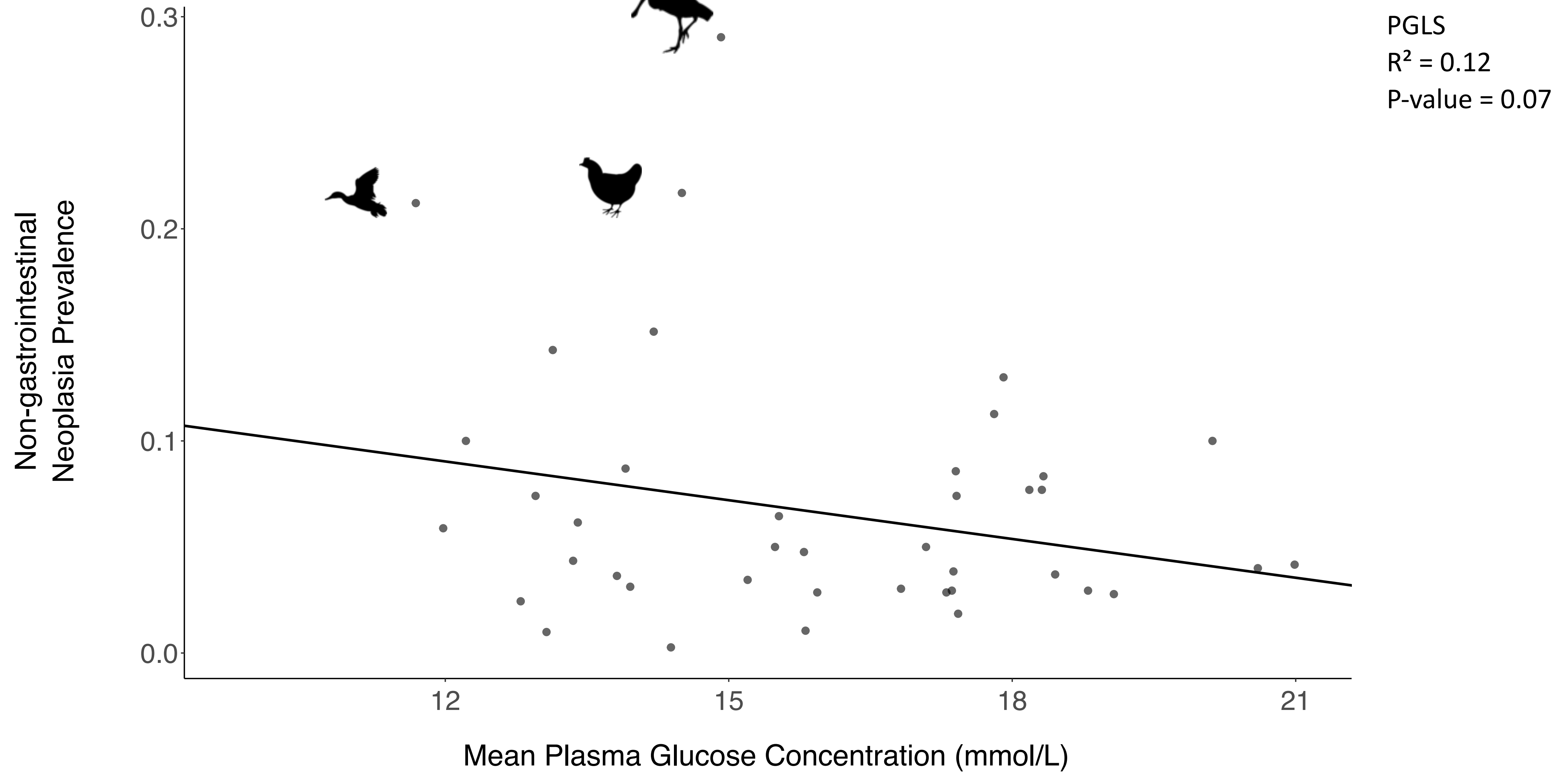

Supplement: Supplement 6 — Supplementary Figure 6. No significant correlation between cancer or neoplasia prevalence and mean plasma glucose concentration in birds. There is no significant correlation between mean plasma glucose concentration and (A) malignancy prevalence across tissues for 51 bird species, (B) neoplasia prevalence across tissues for 51 bird species, (C) gastrointestinal malignancy prevalence for 31 bird species, (D) gastrointestinal neoplasia prevalence for 31 bird species, (E) non-gastrointestinal malignancy prevalence for 41 bird species, and (F) non-gastrointestinal neoplasia prevalence for 41 bird species (PGLS: P-value > 0.05). Each dot shows the malignancy prevalence across tissues (A), the neoplasia prevalence across tissues (B), the gastrointestinal malignancy prevalence (C), the gastrointestinal neoplasia prevalence (D), the non-gastrointestinal malignancy prevalence (E), the non-gastrointestinal neoplasia prevalence (F), and the average plasma glucose concentration of one species. We show images of significant outlier species (Rosner’s test). Animal silhouettes from PhyloPic (http://www.phylopic.org/). [file media-6.pdf]

A

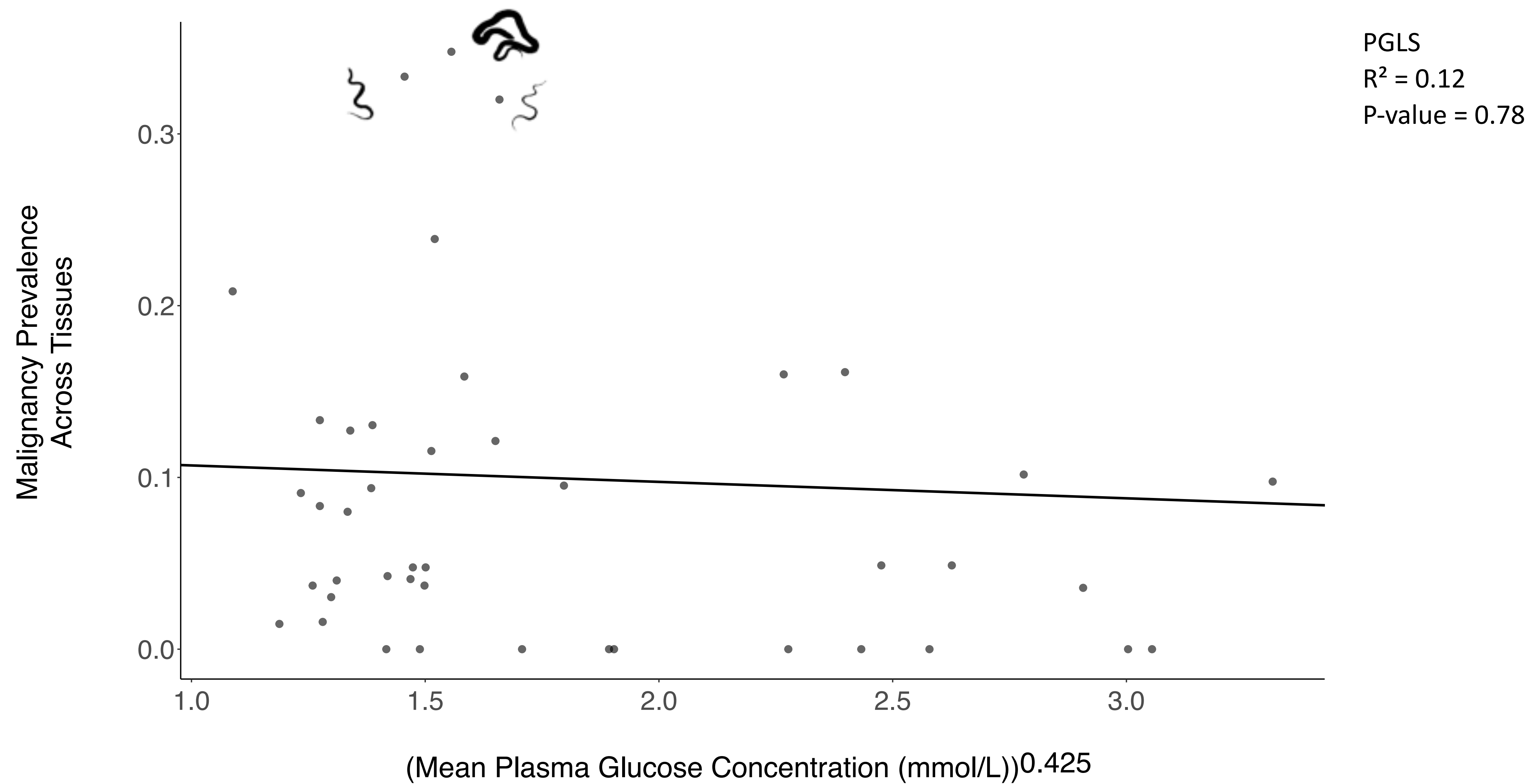

B

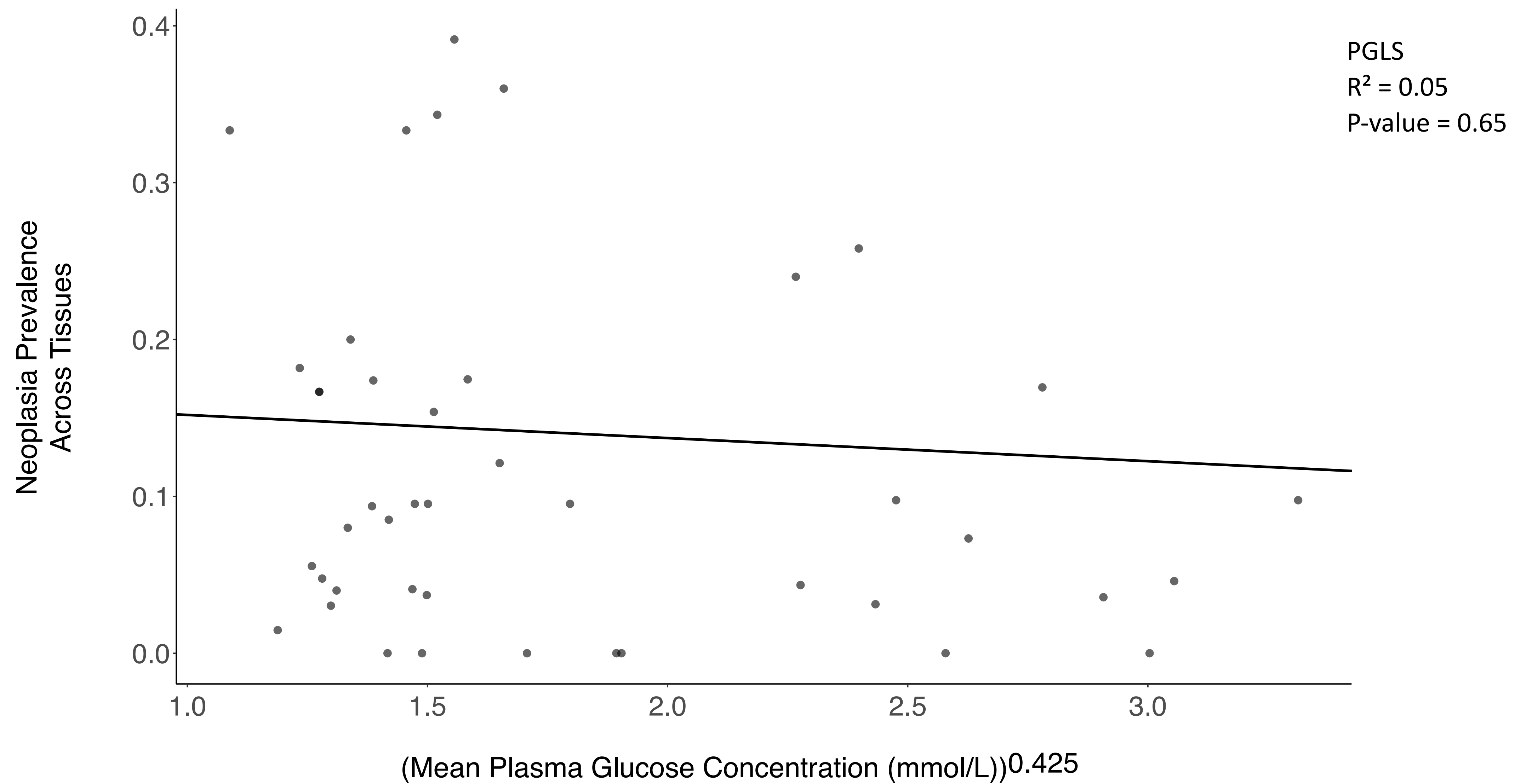

C

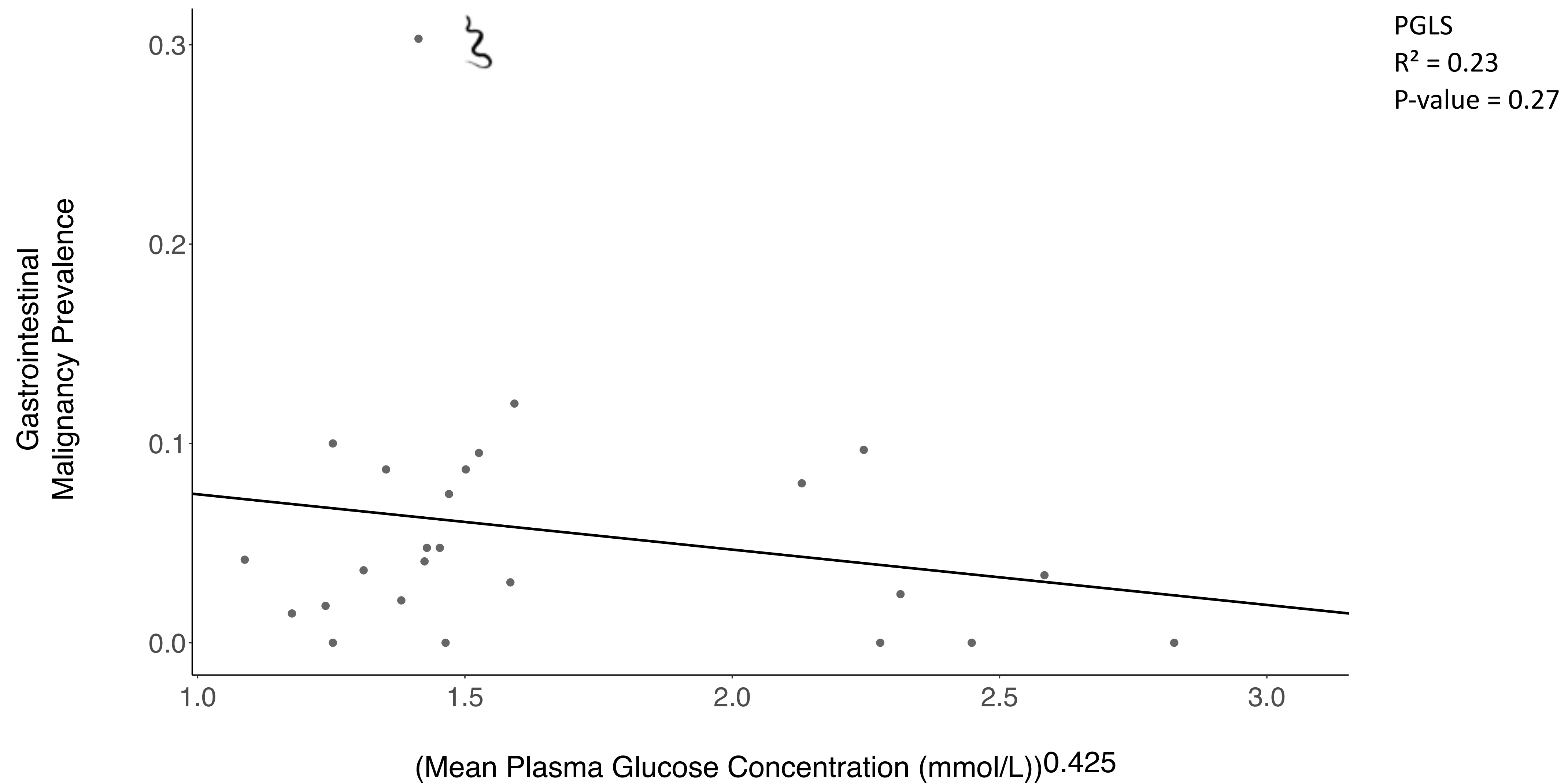

D

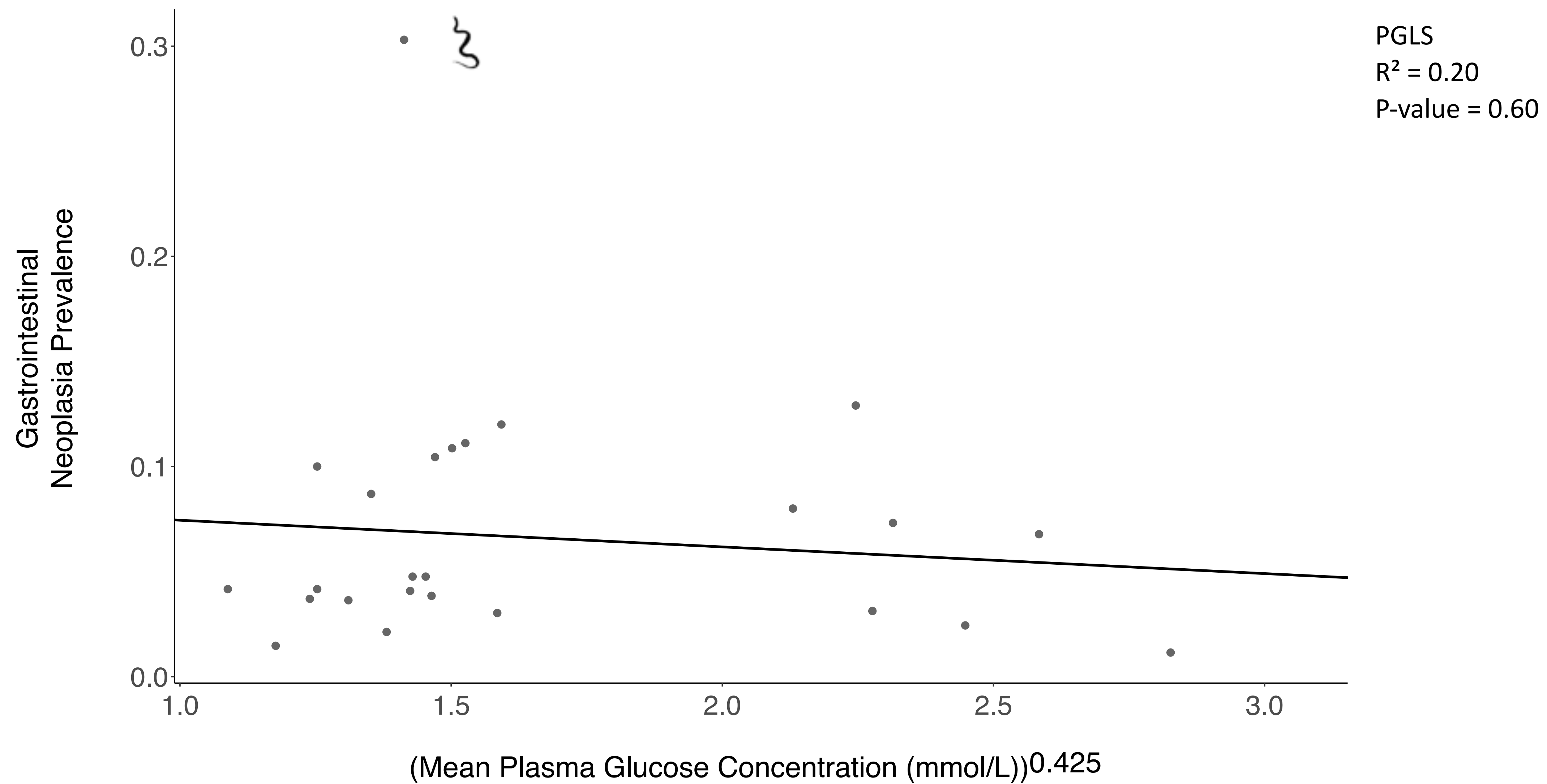

E

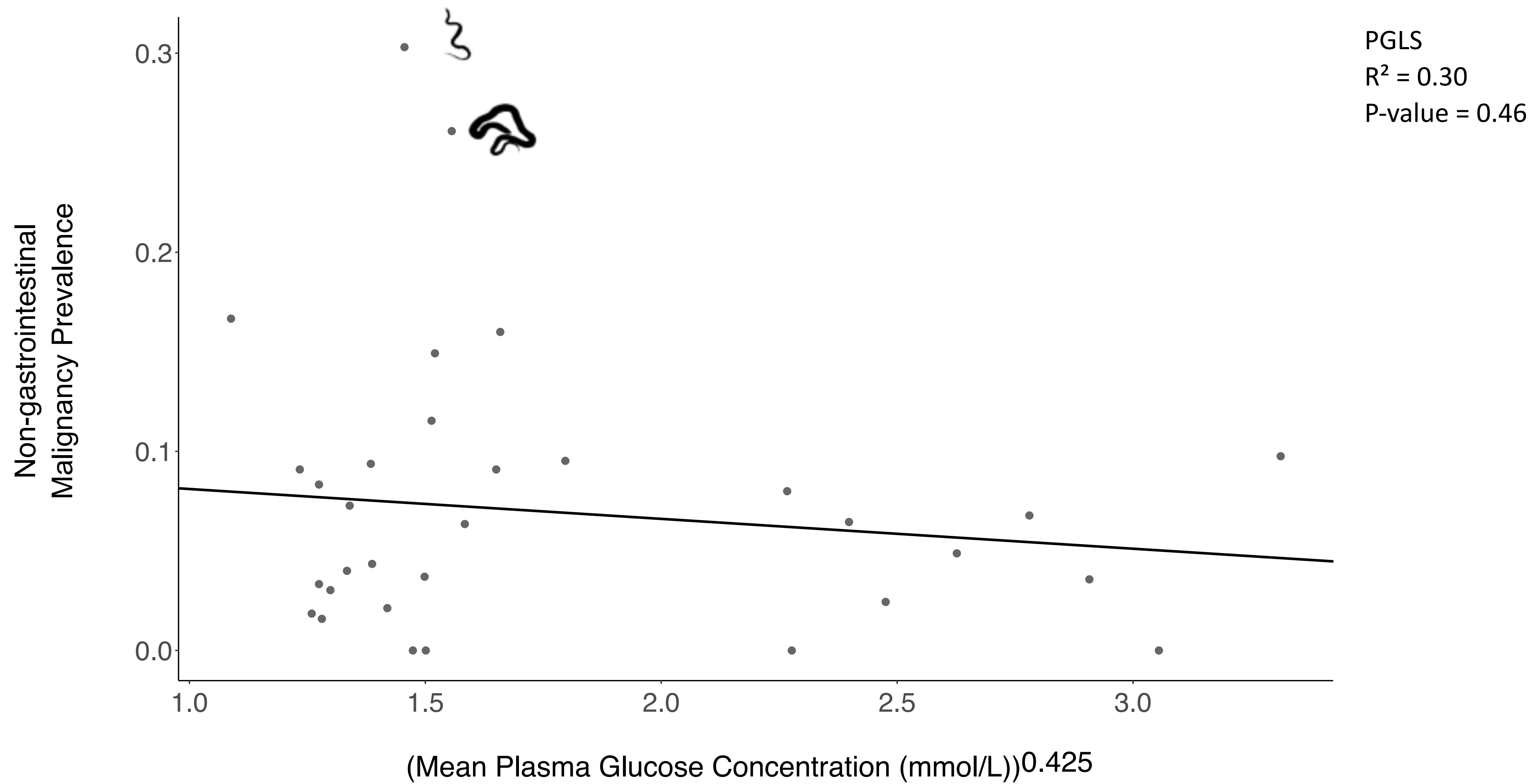

F

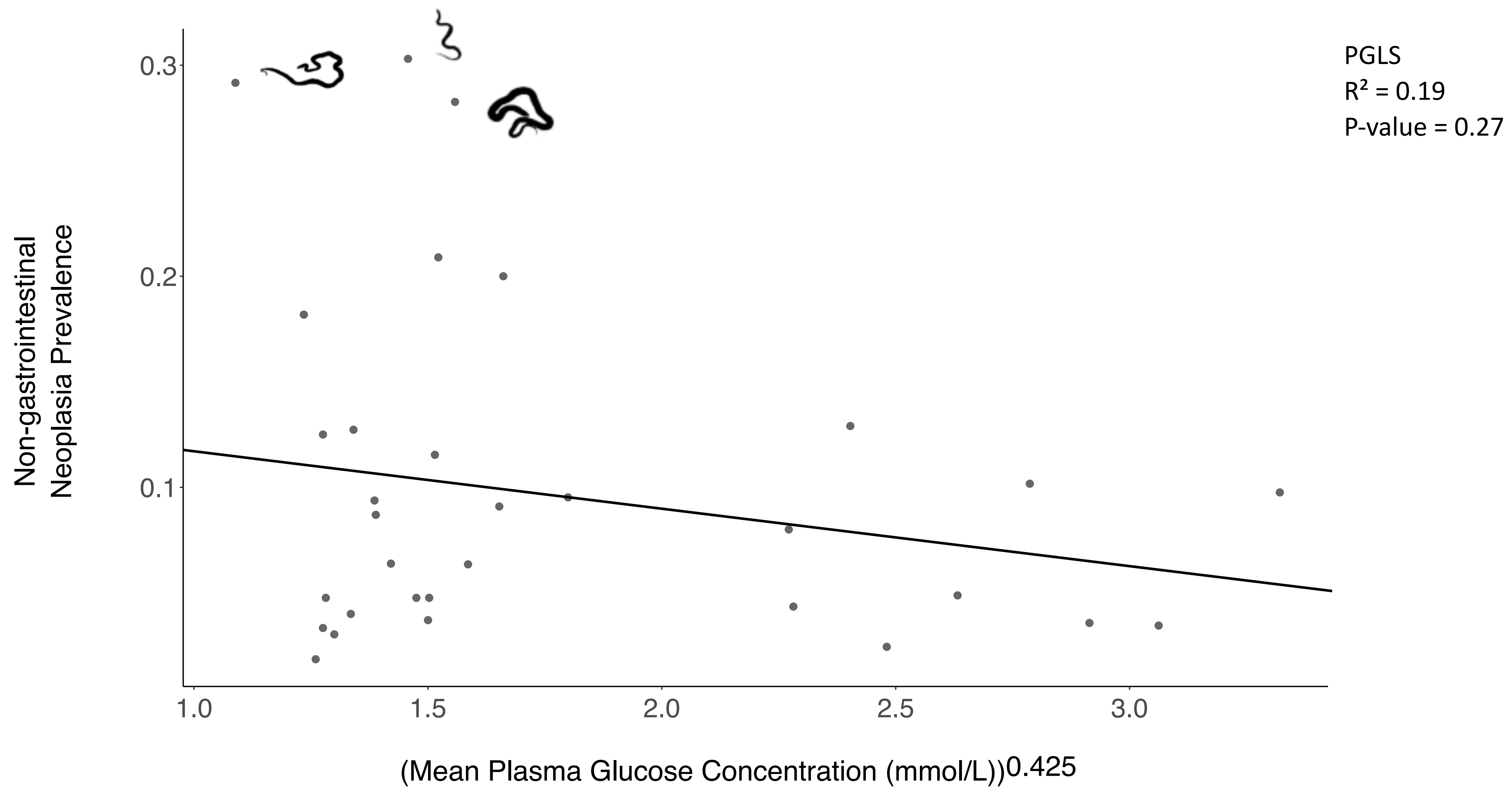

Supplement: Supplement 7 — Supplementary Figure 7. No significant correlation between cancer or neoplasia prevalence and mean plasma glucose concentration in reptiles. There is no significant correlation between mean plasma glucose concentration and (A) malignancy prevalence across tissues for 35 reptile species, (B) neoplasia prevalence across tissues for 35 reptile species, (C) gastrointestinal malignancy prevalence for 25 reptile species, (D) gastrointestinal neoplasia prevalence for 25 reptile species, (E) non-gastrointestinal malignancy prevalence for 32 reptile species, and (F) non-gastrointestinal neoplasia prevalence for 32 reptile species (PGLS: P-value > 0.05). Each dot shows the malignancy prevalence across tissues (A), the neoplasia prevalence across tissues (B), the gastrointestinal malignancy prevalence (C), the gastrointestinal neoplasia prevalence (D), the non-gastrointestinal malignancy prevalence (E), the non-gastrointestinal neoplasia prevalence (F), and the average plasma glucose concentration of one species. We show images of significant outlier species (Rosner’s test). Animal silhouettes from PhyloPic (http://www.phylopic.org/). [file media-7.pdf]
